# The EU-ToxRisk method documentation, data processing and chemical testing pipeline for the regulatory use of new approach methods

**DOI:** 10.1007/s00204-020-02802-6

**Published:** 2020-07-06

**Authors:** Alice Krebs, Barbara M. A. van Vugt-Lussenburg, Tanja Waldmann, Wiebke Albrecht, Jan Boei, Bas ter Braak, Maja Brajnik, Thomas Braunbeck, Tim Brecklinghaus, Francois Busquet, Andras Dinnyes, Joh Dokler, Xenia Dolde, Thomas E. Exner, Ciarán Fisher, David Fluri, Anna Forsby, Jan G. Hengstler, Anna-Katharina Holzer, Zofia Janstova, Paul Jennings, Jaffar Kisitu, Julianna Kobolak, Manoj Kumar, Alice Limonciel, Jessica Lundqvist, Balázs Mihalik, Wolfgang Moritz, Giorgia Pallocca, Andrea Paola Cediel Ulloa, Manuel Pastor, Costanza Rovida, Ugis Sarkans, Johannes P. Schimming, Bela Z. Schmidt, Regina Stöber, Tobias Strassfeld, Bob van de Water, Anja Wilmes, Bart van der Burg, Catherine M. Verfaillie, Rebecca von Hellfeld, Harry Vrieling, Nanette G. Vrijenhoek, Marcel Leist

**Affiliations:** 1 In Vitro Toxicology and Biomedicine, Department Inaugurated by the Doerenkamp-Zbinden Foundation, University of Konstanz, Universitaetsstr. 10, 78457 Konstanz, Germany; 2 Konstanz Research School Chemical Biology, University of Konstanz, 78457 Konstanz, Germany; 3 BioDetection Systems BV, Science Park 406, 1098 XH Amsterdam, The Netherlands; 4 Leibniz-Institut für Arbeitsforschung an der TU Dortmund, Leibniz Research Center for Working Environment and Human Factors (IfADo), Ardeystraße 67, 44139 Dortmund, Germany; 5 Leiden University Medical Center, P.O. Box 9600, 2300 RC Leiden, The Netherlands; 6 Division of Drug Discovery and Safety, Leiden Academic Center for Drug Research, Leiden University, Einsteinweg 55, 2333 CC Leiden, The Netherlands; 7 Edelweiss Connect GmbH, Technology Park Basel, Hochbergerstrasse 60C, 4057 Basel, Switzerland; 8 Aquatic Ecology and Toxicology Group, Center for Organismal Studies, University of Heidelberg, Im Neuenheimer Feld 504, 69120 Heidelberg, Germany; 9 CAAT Europe, University of Konstanz, Steinbeis SU-1866, 78457 Konstanz, Germany; 10 BioTalentum Ltd., Aulich Lajos str. 26, Gödöllő, 2100 Hungary; 11 Simcyp Division, Certara UK Limited, Level 2-Acero, 1 Concourse Way, Sheffield, S1 2BJ UK; 12 InSphero AG, Wagistrasse 27, 8952 Schlieren, Switzerland; 13 Unit of Toxicology Sciences, Swedish Toxicology Sciences Research Center (Swetox), Karolinska Institutet, Forskargatan 20, 151 36 Södertälje, Sweden; 14 Division of Molecular and Computational Toxicology, Department of Chemistry and Pharmaceutical Sciences, Vrije Universiteit Amsterdam, De Boelelaan 1108, 1081 HZ Amsterdam, The Netherlands; 15 Department of Development and Regeneration, Stem Cell Biology and Embryology, Stem Cell Institute Leuven, KU Leuven, O&N IV Herestraat 49, 3000 Leuven, Belgium; 16 Department of Experimental and Health Sciences, Research Programme on Biomedical Informatics (GRIB), Institut Hospital del Mar d’Investigacions Mèdiques (IMIM), Universitat Pompeu Fabra, 08003 Barcelona, Spain; 17 European Molecular Biology Laboratory, European Bioinformatics Institute (EMBL-EBI), Wellcome Genome Campus, Cambridge, UK; 18 Leiden Academic Center for Drug Research, LACDR/Toxicology, Leiden University, PO Box 9500, 2300 RA Leiden, The Netherlands; 19 Switch Laboratory, Department of Cellular and Molecular Medicine, VIB‐KU Leuven Center for Brain and Disease Research, KU Leuven, Herestraat 49, 3000 Leuven, Belgium; 20 trenzyme GmbH, Byk-Gulden-Str. 2, 78467 Konstanz, Germany; 21 Department of Biochemistry and Biophysics, Stockholm University, 10691 Stockholm, Sweden

**Keywords:** GIVIMP, In vitro toxicology, Nuclear receptor, Metadata, Data processing

## Abstract

Hazard assessment, based on new approach methods (NAM), requires the use of batteries of assays, where individual tests may be contributed by different laboratories. A unified strategy for such collaborative testing is presented. It details all procedures required to allow test information to be usable for integrated hazard assessment, strategic project decisions and/or for regulatory purposes. The EU-ToxRisk project developed a strategy to provide regulatorily valid data, and exemplified this using a panel of > 20 assays (with > 50 individual endpoints), each exposed to 19 well-known test compounds (e.g. rotenone, colchicine, mercury, paracetamol, rifampicine, paraquat, taxol). Examples of strategy implementation are provided for all aspects required to ensure data validity: (i) documentation of test methods in a publicly accessible database; (ii) deposition of standard operating procedures (SOP) at the European Union DB-ALM repository; (iii) test readiness scoring accoding to defined criteria; (iv) disclosure of the pipeline for data processing; (v) link of uncertainty measures and metadata to the data; (vi) definition of test chemicals, their handling and their behavior in test media; (vii) specification of the test purpose and overall evaluation plans. Moreover, data generation was exemplified by providing results from 25 reporter assays. A complete evaluation of the entire test battery will be described elsewhere. A major learning from the retrospective analysis of this large testing project was the need for thorough definitions of the above strategy aspects, ideally in form of a study pre-registration, to allow adequate interpretation of the data and to ensure overall scientific/toxicological validity.

**Electronic supplementary material:**

The online version of this article (10.1007/s00204-020-02802-6) contains supplementary material, which is available to authorized users.

## Introduction

Animal-free new approach methods (NAM) are increasingly used for the characterization of chemical hazards. This makes it necessary to define the conditions, under which the information from such assays can be considered ‘valid’, i.e. robust, reproducible, transparent and linked to a set of measures of uncertainty at all levels of data generation.

Hundreds of NAM are available to researchers, some highly complex, such as microphysiological systems (Marx et al. 2016), others being inexpensive and allowing high throughput (Adler et al. 2011; Bal-Price et al. 2018; Judson et al. 2017; Leist et al. 2012b; Liu et al. 2017; Richard et al. 2016; Zimmer et al. 2012). However, the assembly of such NAM to batteries is demanding, and the use across multiple laboratories in coordinated research activities is particularly challenging (Aschner et al. 2017; Behl et al. 2015, 2019; Jacobs et al. 2016; Jaworska et al. 2015; Judson et al. 2017; Legradi et al. 2018; Li et al. 2017; Sonneveld et al. 2011; Thomas et al. 2019).

Current regulatory procedures are mostly based on in vivo guideline studies, such as the OECD test guidelines 424 (OECD 1997), 426 (OECD 2007), 411 (OECD 1981), or 451 (OECD 2018b) on neurotoxicity, developmental neurotoxicity, sub-chronic toxicity (90 days) or carcinogenicity, respectively. Besides limitations in throughput, it is becoming more and more evident that animal-based hazard evaluation may not only yield false negatives (FN) endangering human health (Grass and Sinko 2002; Leist and Hartung 2013; Luechtefeld et al. 2018; Olson et al. 2000; Wang and Gray 2015), but also produces many false positives (FP) leading to large technological and economic losses (Hartung and Leist 2008; Hartung and Rovida 2009; Meigs et al. 2018). The increased use of NAM would probably remedy some of these problems (Collins et al. 2008; Hsieh et al. 2019; Leist et al. 2008b; Tice et al. 2013). However, most of the available methods do often not fulfill the requirements of regulators, as their technical background, reliability, and predictivity are not well documented.

The International STakeholder NETwork consortium (ISTNET) has designed a questionnaire that scores the readiness level of a NAM for regulatory purposes (Bal-Price et al. 2018). This needs further testing and refinement to be broadly applicable. Furthermore, the assessment of the reliability of alternative methods for regulatory purposes should also include rapidly developing new technologies (e.g. induced pluripotent stem cells, 3D cell co-cultures and organoids, high-content omics measurements, bioinformatics tools, etc.) (Leist et al. 2008a, 2014; Marx et al. 2016; Pamies et al. 2018; Rovida et al. 2015; Rusyn and Greene 2018; Schmidt et al. 2017; Smirnova et al. 2016).

For the regulatory use of data from NAM, four aspects of data generation are important: (i) description of the test method and its performance, (ii) transparent data processing and storage, (iii) documentation of the test compounds, and (iv) procedures for the use of the data in the context of integrated approaches to testing and assessment (IATA). This latter aspect also implies in vitro to in vivo extrapolation (IVIVE) and biological interpretation of NAM data. Several large-scale cooperative projects have improved our understanding of the above aspects of how remaining gaps may be filled, as exemplified below:

ReProTect was a consortium set up by the European Center for the Validation of Alternative Methods (ECVAM) to develop a testing strategy for reproductive toxicity (Hareng et al. 2005). This project recognized the need for standard operating procedures (SOPs) to be deposited in a public database, DB-ALM (Roi 2006). Moreover, a feasibility study with blinded testing of ten chemicals in 14 assays evaluated the overall performance of the test battery (Schenk et al. 2010).

The AcuteTox project aimed to demonstrate that animal tests for acute systemic toxicity can be replaced by NAM. This project pioneered inter-laboratory data and method storage and it explored test battery optimization. High-level statistical approaches were used to define optimum test combinations, taking human data as reference. Also, test compound handling (dissolution, storage) was standardized across many partners (Clemedson et al. 2007; Clothier et al. 2008; Clothier 2007; Kinsner-Ovaskainen et al. 2009, 2013).

The ESNATS (Embryonic Stem cell-based Novel Alternative Testing Strategies) project developed a test battery based on human embryonic stem cells (hESCs) (Rovida et al. 2014). This initiative further developed the description of a tiered screening strategy and also exemplified the documentation of test compounds (Zimmer et al. 2014). Assays resulting from the project demonstrated how omics technologies may be used in a quantitative way for toxicological prediction models (Pallocca et al. 2016; Rempel et al. 2015; Shinde et al. 2015, 2016, 2017; Waldmann et al. 2017).

The ToxCast program is yet the largest chemical screening project with information from more than 1000 high-throughput assay endpoints and a very broad scope. They addressed important aspects like the automated analysis of data, and the building of algorithmic pipelines to arrive at summary test data (AC_50_ values). Moreover, comprehensive NAM data interpretation was anchored and calibrated against available animal data. More recently, this project also showed ways of how to link NAM data to human exposure levels by IVIVE (Bell et al. 2018; Casey et al. 2018; Wambaugh et al. 2018; Wetmore et al. 2014, 2015).

Test validation and regulatory acceptance were important aspects of the ChemScreen project (van der Burg et al. 2015b), and a central role was taken by the CALUX^®^ assays. These tests had been prevalidated in the context of ReProTect (van der Burg et al. 2010a, b), and some were subsequently validated by the OECD and ECVAM. These cell-based reporter assays quantify chemical interactions with various nuclear receptors. Their readout was combined with in silico information and absorption, distribution, metabolism and excretion (ADME) predictions for toxicological hazard assessment (Bosgra and Westerhout 2015).

The EU-ToxRisk project profited from the above and other research initiatives in further defining the requirements for collaborative testing. The consortium of 39 partners from academia, industry and regulatory authorities is funded by the European Commission with the goal to establish new animal-free strategies of hazard evaluation. These new concepts comprise in vitro methods, based exclusively on human cells, as well as in silico methods like read-across and quantitative structure–activity relationship (QSAR) (Daneshian et al. 2016; Delp et al. 2019; Graepel et al. 2019; Nyffeler et al. 2018).

As EU-ToxRisk has a strong focus on the regulatory acceptance of its strategy, a case study was designed to establish, test and validate all processes required to make NAM acceptable in legal contexts of data submission. This cross-systems testing study, based on 19 well-characterized chemicals and > 20 test methods, was used to define and standardize all different aspects of NAM-based testing in a large research consortium. For instance, method documentation was established, taking into account the Guidance Document on Good In Vitro Method Practices (GIVIMP) (OECD 2018a), good cell culture practice (GCCP) (Coecke et al. 2005; Hartung et al. 2002), the OECD guidance document 211 on non-guideline methods (OECD 2017), and more general previous recommendations on test documentation (Leist et al. 2010; Schmidt et al. 2017; Zimmer et al. 2012). We established data formats and processing pipelines, characterized the robustness, sensitivity and throughput of the methods, and data formats, as well as processing pipelines. In the present communication, we disclose the resulting optimized guidance and processes, and we give examples of their use, to allow their implementation in future collaborative research consortia.

## Materials and methods

### Test compounds

Test compounds were distributed to project partners by the Joint Research Center (JRC). Shipping and storage were according to the manufacturers’ instructions. Stock solutions were prepared by the individual partners in dimethyl sulfoxide (DMSO), phosphate buffered saline (PBS), water or culture medium, according to centralized instructions. Detailed information about the compound supplier and catalog number is provided in Suppl. Fig. SM_1. Compound aliquots of 10 µl each were stored at − 80 °C until use. Paraquat was always dissolved freshly in cell culture medium at the desired concentration prior to each use. The final DMSO concentration was 0.1% under all test conditions (any compound at any concentration). Documentation of the physicochemical properties were derived using the ChemAxon software (Budapest, Hungary). To calculate the logK, i.e. the log_10_ K_ow_ (K_ow_: octanol/water partition coefficient), the software uses the method described by Viswanadhan et al. (1989). Aqueous solubility of compounds was predicted using ChemAxon's Solubility Predictor, which uses a fragment-based method that identifies different structural fragments in the molecule and calculates their solubility contribution. The algorithm is described by Hou et al. (2004).

### Determination of free compound concentration in cell culture media

*Lipid and protein in medium*: The concentrations of lipid (mg/ml) and protein (µM) in cell culture media were extracted from the EU-ToxRisk test method descriptions and SOPs. Protein concentration expressed as mg/ml in the test methods was converted to µM assuming a molecular weight of 66.5 kD for bovine albumin, and assuming that albumin represents well all other serum proteins (assuming 1 Da = 1 g/mole). In those test methods to which fetal calf serum (FCS) was added, the final protein concentration in the media containing FCS was calculated, based on the reference value of 23 mg/ml reported for commercial FCS used in medium supplementation (Lindl 2002). The amount of FCS used in the test methods was reported to have been either 5 or 10% in the medium.

*Plasma protein binding (PPB)*: The plasma protein binding values for drugs (colchicine, valproate, clofibrate, hexachlorophene, ibuprofen, paracetamol, rifampicin, paclitaxel, tolbutamide) were extracted from the DrugBank database (Wishart et al. 2006). The PPB of sulfisoxazole was extracted from the toxicology data network (TOXNET) of the US national library of medicine. Values for carbaryl, rotenone, tebuconazole, triphenyl phosphate and acrylamide were from the chemistry dashboard of the US environmental protection agency (EPA). All values were experimentally determined, except for acrylamide which was a predicted value (U.S. Environmental Protection Agency. Chemistry Dashboard. https://comptox.epa.gov/dashboard/DTXSID5020027 (accessed January 20, 2020). The value for mercuric chloride was extracted from the book of Nordlind (1990), while that of polychlorinated biphenyl 180 (PCB 180) was reported by Brown and Lawton (1984). The PPB value of paraquat was reported in the forensic examination by Houze et al. (1990).

*Free concentrations in complete medium*: To predict the test compounds’ free (unbound) fraction in the treatment medium, it was necessary to account for the binding components in the medium. This was based on the following assumptions: (i) binding to albumin and lipid tri-acyl glycerol (TAG) in complete culture media are the only significant processes limiting the availability of free test compound; (ii) the binding to protein and lipid in culture media is linear within the tested concentration range; (iii) compounds with an air–water partition coefficient (*K*_AW_ < 0.03) were considered non-volatile. This assumption was found earlier (Fischer et al. 2017) to apply for 95% of the investigated compounds. Note that HgCl_2_ (*K*_AW_ = 0.02) may be a borderline compound (Sommar et al. 2000). (iv) Binding to plastics used in cell culture is not considered in this prediction of free fraction of test compounds. This condition applies strictly only if plastic is pre-adsorbed with test chemicals. This approach was applied here, e.g. for the zebrafish assay. Plastic binding data would otherwise require experimental assessment, as their prediction has large uncertainties. To indicate the range of deviation, data have been obtained for PCB180, one of the most hydrophobic and plastic-binding compounds of the test chemicals—and about one third of the compound was bound to plastic (Nyffeler et al. 2018). As most tests used similar cell culture dishes (96-well), we assumed that plastic binding did not largely affect the comparability of test results of a given chemical between laboratories. The maximal tested concentration did not exceed the solubility of the compound in complete culture medium.

### Test methods

**Fig. 1:**
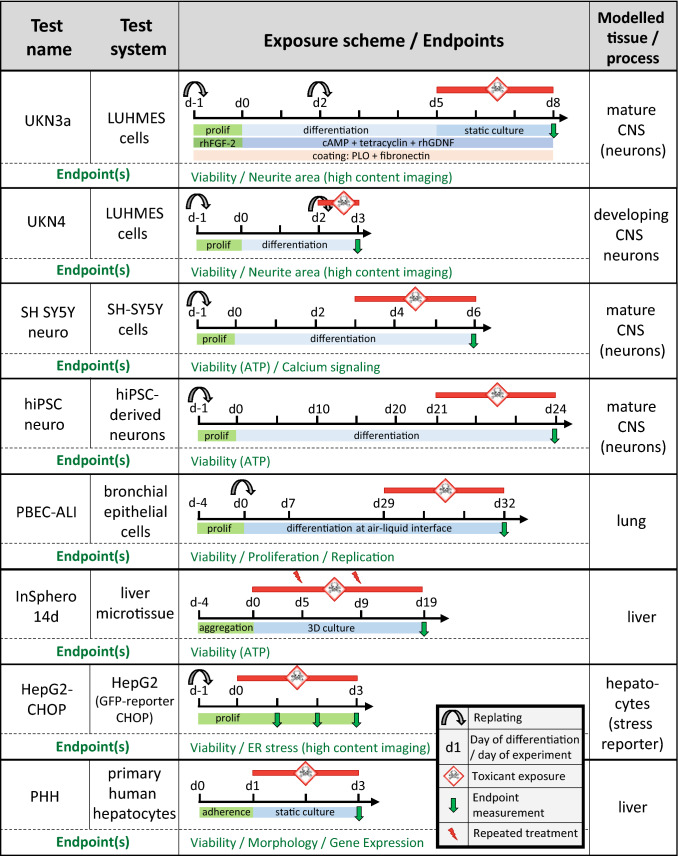
Exposure schemes of representative test methods as part of the test method description. A generic symbol language to display exposure schemes has been developed. Eight methods were chosen for exemplary display, while all others can be found in Suppl. Fig. 1. Information is given on the test system (type of cells used), and its treatment before and during execution of the test. The time axes displayed show the pivotal culture period determining the experimental outcome, displayed in units of days (**d**). The period of compound exposure is highlighted in red, with the flash arrow symbol indicating when test compound is re-added. The green and blue bars give general information on the culture state (e.g. proliferation (prolif) or adherence phase). In a more complete version of the graphical scheme (exemplified here for UKN3a only), additional information layers on cell medium additives and type of plastic coating would also be given (color figure online)

**Fig. 2:**
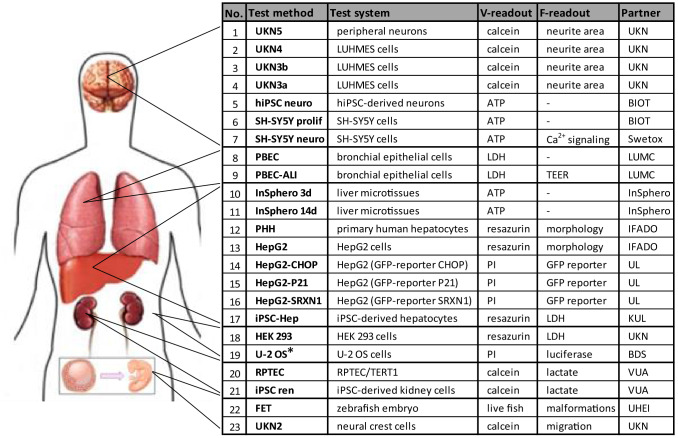
Overview of the panel of test methods used to assess repeated dose toxicity to key organs (RDT) and developmental toxicity (DART). The cross-systems testing case study of EU-ToxRisk comprised 23 test method families using 18 different test systems. For instance test method family No. 19, U-2 OS, comprised 25 different reporter assays (CALUX^®^ assays)*, using luciferase expression in U-2 OS as measure of nuclear receptor modulation and other signaling pathways. The test method family No. 7 could be run as viability test method or as functional method examining Ca^2+^ signals triggered by opening of voltage-operated calcium channels. The test systems represent important features of the human nervous system, lung, liver, and kidney. Some systems (No. 18 and No. 19) representing less specialized cell types were included as potential negative controls of tissue specificity. Cells relevant for developmental and reproductive toxicity (DART) assessment were also included (No. 22 and No. 23). The assays were performed in 11 different laboratories. Besides viability (primary V-readout), often (i.e. in 16 of the 23 test methods) a functional readout (secondary F-readout) was also assessed. The contributing institutions were: UKN = University of Konstanz (D); BIOT = BioTalentum (HU); Swetox (SE). LUMC = Leiden University Medical Center (NL); InSphero GmbH (CH); IfADo at the Technical University Dortmund (D); UL = University of Leiden (NL); KUL = Catholic University of Leuven (BE); VUA = Free University Amsterdam (NL); UHEI = University of Heidelberg (D); BDS = BioDetection Systems (NL). TEER = Transepithelial electrical resistance

Out of the 23 test methods (method families), 22 were based on human cells. The fish embryo toxicity (FET) test is based on zebrafish (*Danio rerio*) embryos. Schematic representations of eight exemplary test method exposure schemes are given in Fig. 1; the schematic depiction of all test methods can be found in Suppl. Fig. SM_2. An overview table of all tests and their literature references is compiled in Suppl. Tab. SM_3. An overview of test readouts and of the participating laboratories is provided in Fig. 2. In addition, a public database of test descriptions was established (https://eu-toxrisk.douglasconnect.com/public/). Therefore, only brief overviews of the tests are given below.

*UKN5 (PeriTox):* The assay is based on immature human dorsal root ganglia neurons differentiated from pluripotent stem cells as described in detail earlier (Hoelting et al. 2016). After thawing of pre-differentiated neurons, these were seeded to multi-well plates and treated with test compounds for 24 h. To assess cell viability and neurite area by high-content imaging, the cells were stained with calcein-AM and Hoechst H-33342.

*UKN4 (NeuriTox):* LUHMES neuronal precursors were differentiated for two days, before they were exposed to test compounds for 24 h. Cell viability and neurite area were measured by high-content imaging on day 3 of differentiation (d3) (Delp et al. 2018, 2019; Krug et al. 2013). A detailed SOP is available at the ECVAM DB-ALM database (protocol No. 200).

*UKN3b:* In this variant of the NeuriTox test, LUHMES cells were differentiated for 5 days to obtain mature neurons (Lotharius et al. 2005; Scholz et al. 2011). These were exposed to test compounds for 24 h. To assess cell viability and neurite area by high-content imaging after treatment on d6, the cells were stained with calcein-AM and Hoechst H-33342 (Krug et al. 2013). A detailed SOP is available at the ECVAM DB-ALM database (protocol No. 196).

*UKN3a:* The method is similar to UKN3b (see above), however cells were exposed to compounds for 72 h, from d5 until d8. A detailed SOP of the method is available at the ECVAM DB-ALM database (protocol No. 202).

*hiPSC neuro:* Human iPSC line SBAD2 was used to derive neuronal precursor cells (NPCs). These were differentiated to mixed cortical type neurons and glial cultures for 21 or 42 days. After 72 h of test compound exposure, the viability was assessed by an ATP assay. A detailed SOP is available at the ECVAM DB-ALM database (protocol No. 208 and 207).

*SH-SY5Y prolif:* SH-SY5Y cells were seeded to multi-well plates, and medium was changed to proliferation medium containing test compound at 24 h after seeding. After 72 h of compound exposure, the viability of cells was determined, using their ATP content as an endpoint. A detailed SOP is available at the ECVAM DB-ALM database (protocol No. 210).

*SH-SY5Y neuro:* Proliferating SH-SY5Y neuroblastoma cells were differentiated for 3 days to semi-mature neurons by exposure to retinoic acid (RA). The cells were subsequently exposed to test compounds for 72 h in the continued presence of RA. On d6, the ATP content was determined and calcium signaling was assessed by measurement of basal intracellular Ca^2+^ levels and activation of voltage-dependent Ca^2+^ channels (induced by exposure to 30 mM KCl). Detailed SOPs are available at the DB-ALM database (ATP assay protocol ECVAM DB-ALM No. 205 and Calcium assay protocol ECVAM DB-ALM No. 206).

*PBEC:* Primary human bronchial epithelial cells (PBEC) were seeded into conventional multi-well plates (without transwell inserts) and exposed to compound for 72 h.

*PBEC-ALI:* Primary human bronchial epithelial cells were seeded into transwell tissue culture inserts and grown submerged. The medium above the confluent cell layer was removed after 7 days followed by differentiation at the air–liquid interface for 22 days. These mature PBEC-ALI cultures were exposed to test compounds in their medium for 72 h. Toxicity was assessed by the release of LDH (Boei et al. 2017; van Wetering et al. 2000). Transepithelial electrical resistance (TEER) was measured as functional endpoint.

*InSphero 3d:* Primary human hepatocytes (PHH) were used to produce liver microtissues, using established InSphero organo plate technology (Kijanska and Kelm 2004; Messner et al. 2013). After four days of aggregation, microtissues were exposed to test compounds for three days. Viability was determined by their ATP content.

*InSphero 14d:* The method is similar to ‘InSphero 3d’ (see above), but test compound exposure was prolongued to 14 days, with re-dosing on days 5 and 9 after initial treatment.

*PHH:* Primary human hepatocytes of single donors (lot data available via co-author W. Albrecht) were seeded to multi-well plates after thawing. One day after seeding, cells were exposed to test compounds for 48 h. The viability was measured by resazurin reduction.

*HepG2:* HepG2 cells were exposed to test compounds for 48 h. Viability was assessed by resazurin reduction.

*HepG2 reporter (HepG2-CHOP, HepG2-P21, HepG2-SRXN1):* stable stress response reporter cell lines were engineered to express GFP-reporter constructs under the control of natural promoters (on a bacterial artificial chromosome) of SRXN1 (for oxidative stress), P21 (for DNA damage) and CHOP (for ER stress response). Cell count (Hoechst staining H-33342), pathway induction (GFP intensity) and cell viability (propidium iodide staining) were assessed at 24 h, 48 h and 72 h after test compound exposure by high content imaging (Schimming et al. 2019; Wink et al. 2017, 2018).

*iPSC-Hep:* iPSCs cells were grown on matrigel-coated plates, and a 30-day differentiation protocol towards the hepatocyte lineage was commenced when the cells reached 70–80% confluency (Vanhove et al. 2016). The viability of the differentiated hepatocytes after 24 h of compound exposure was determined by a resazurin reduction assay.

*HEK 293:* These relatively de-differentiated cells from fetal kidney grow as epithelioid monolayers. They were seeded to multi-well plates and exposed to test compounds for 24 h. Cell viability was subsequently assessed by measurement of resazurin reduction and release of lactate dehydrogenase (LDH). A detailed SOP is available at the ECVAM DB-ALM database (protocol No. 201).

*U-2 OS cells:* These osteosarcoma cells are relatively de-differentiated and grow in an epitheloid way. Their viability was assessed based on constitutive luciferase expression (van Vugt-Lussenburg et al. 2018) in the context of the automated CALUX^®^ reporter gene assay procedure (see paragraph below). A detailed SOP is available at the ECVAM DB-ALM database (protocol No. 197).

*RPTEC:* RPTEC/TERT1 immortalized kidney proximal tubule cells (Wieser et al. 2008) were used at 7 days after confluence (i.e. differentiated, non-proliferative state) (Aschauer et al. 2013). Monolayers were exposed to test compounds for 24 h. Toxicity was assessed by quantitation of resazurin reduction capacity, calcein-AM uptake and quantification of lactate production (Limonciel et al. 2011).

*iPSC ren:* Proximal tubular-like cells (PTL) were differentiated from iPSC (SBAD2 clone 1). On day 16 of differentiation (contact Dr. Wilmes, VUA for protocol). Cells were passaged into 96-well plates, cultured to confluence, and stabilized for an additional 7 days. Cells were then exposed to test compounds for 24 h. Toxicity was assessed by quantitation of resazurin reduction capacity, calcein-AM uptake and quantification of lactate production.

*FET:* Fertilized zebrafish (*Danio rerio;* west aquarium strain) eggs were exposed to test compounds at 1.5 h post fertilization (hpf). Several morphological endpoints were scored at 96 hpf. All technical details have been described earlier (Braunbeck et al. 2015) and are given in OECD TG 236 (OECD 2013). A detailed SOP is available at the ECVAM DB-ALM database (protocol No. 140).

*UKN2 (cMINC):* Pre-differentiated neural crest cells (NCC) (Zimmer et al. 2012) were seeded to coated multi-well plates with inserted silicon stoppers to create a cell-free area as described earlier (Nyffeler et al. 2017a, b). Cell migration was initiated one day after seeding by removal of the stopper, and test compound was added. Migration was assessed after 24 h of compound exposure by high content imaging. A detailed SOP is available at the ECVAM DB-ALM database (protocol No. 195).

### CALUX^®^ assays

*Cell lines and cell culture*: The CALUX^®^ (Chemically Activated LUciferase eXpression) cell lines as described by Sonneveld et al. (2005) are human U-2 OS osteosarcoma cells each stably transfected with an expression construct for various human receptors, and a reporter construct consisting of multimerized responsive elements for the cognate receptor or cell signaling pathway coupled to a minimal promoter element (TATA) and a luciferase gene. Cells were maintained as described previously (Sonneveld et al. 2005). The Cytotox CALUX^®^, used as a control line for non-specific effects, consists of human U-2 OS cells stably transfected with an expression construct constitutively expressing the luciferase gene, and is described in (van der Linden et al. 2014). Wild-type U-2 OS cells (HTB-96) were obtained from ATCC. Also part of the panel was the AhR CALUX^®^ assay, based on rat hepatoma H-4-II-E cells (ATCC CRL-1548); this cell line is described in detail in (Garrison et al. 1996) under the name DR CALUX^®^.

*CALUX*^*®*^* assay procedure*: Testing was performed in non-blinded fashion. The automated CALUX^®^ assays were carried out as described earlier (van der Burg et al. 2015a). In brief, the assay was performed in assay medium, consisting of DMEM without phenol red indicator (Gibco) supplemented with 5% charcoal-stripped fetal calf serum (DCC), 1 × non-essential amino acids (Gibco) and 10 U/ml penicillin and 10 µg/ml streptomycin. A cell suspension in assay medium was made of 1 × 10^5^ cells/ml, and white 384-wells plates were seeded with 30 µl cell suspension/well. After 24 h, exposure medium was prepared. A dilution series in 0.5 log unit increments of each test compound (in DMSO) was added to a 96-wells plate containing assay medium. Of this exposure mixture, 30 µl was added to the assay plates containing the CALUX^®^ cells, resulting in a final DMSO concentration of 0.1%. Additionally, DMSO blanks and a full dose response curve of the reference compounds were included on each plate. All samples were tested in triplicates. The preparation of the compound dilution series as well as the exposure of the cells were performed on a Hamilton Starlet liquid handling robot coupled to a Cytomat incubator. After 24 h, the exposure medium was removed using an EL406 washer-dispenser (BioTek) and 10 µl/well triton lysis buffer (25 mM Tris, 2 mM DTT and 2 mM EDTA in demineralized water, with 10% (v/v) glycerol and 1% (v/v) Triton^®^ X-100, pH adjusted to 7.8) was added by the EL406. Subsequently, the luciferase signal was measured in a luminometer (InfinitePro coupled to a Connect Stacker, both TECAN). To be able to detect receptor antagonism, the assays were also performed in antagonistic mode using the receptor cell lines. The assay procedure was as described above, with the only exception that the reference agonists were present during the exposure at a concentration corresponding to their EC_50_. Detailed information about reference compounds for each assay can be found in Suppl. Fig. SM_4. Information on the calculation of assay summary data, and their exact definition is compiled in Suppl. Fig. SM_4.

### Test method documentation

The EU-ToxRisk consortium created a detailed test method description template to complement the Standard Operating Procedure (SOP), which was adopted from the EU Reference Laboratory for alternatives to animal testing (ECVAM; https://ecvam-dbalm.jrc.ec.europa.eu/). While the SOP focuses on practical and experimental aspects, the test method documentation was designed to give all information on methods that is relevant to judge the uncertainties of this method and to evaluate if and how the data can be used for risk assessment. The SOPs have been deposited at the DB-ALM database (https://ecvam-dbalm.jrc.ec.europa.eu/methods-and-protocols). An overview of the content of the test method description template has been recently published (Krebs et al. 2019b) and public access to the test method description is possible under https://eu-toxrisk.douglasconnect.com/public/.

### Test method data base

All test methods applied in the EU-ToxRisk project have been documented and are publicly accessible on the test method repository (https://eu-toxrisk.douglasconnect.com/public/). To guide the user through the progress of creating a test method description, a web interface was created for internal use in the EU-ToxRisk project. The web-based guidance has been compiled and will be made publicly available in due course, while the printed version is already available now (Krebs et al. 2019b). All submitted test methods were reviewed by the project’s quality assurance group, and often several rounds of amendments followed. Only accepted versions were made public. Revisions and changes can be entered by the registered user on the repository. A ‘version management system’ has been implemented, as test methods often evolve, as important materials, chemicals and instrumentation change.

### Readiness evaluation

The test method readiness was assessed on the basis of the first version of the test method description created by the EU-ToxRisk consortium (accessible at https://eu-toxrisk.douglasconnect.com/public/). Information from SOPs, deposited at DB-ALM (https://ecvam-dbalm.jrc.ec.europa.eu/methods-and-protocols), was added where available. The items, criteria and respective maximum scores for evaluation of test readiness were used exactly as described in (Bal-Price et al. 2018). Two experts evaluated the test methods independently of each other, and scored each aspect based on available documentation. Then the average of the two scorings was calculated for each sub-item. All scores of the sub-items of the 13 main aspects were added up, and the sum was expressed as percentage of maximum points reachable. A classification scheme was used to summarize the results as high readiness (100–85%; green), intermediate readiness (85–50%; orange) and low readiness (< 50%; red).

### Data storage

The BioStudies database (Sarkans et al. 2018) was used as data warehouse for data generated within the EU-ToxRisk project. All datasets were strictly and unseparatably linked to corresponding assay information in the test method descriptions. The integration of the EU-ToxRisk test method repository and the BioStudies database into one common platform, the EU-ToxRisk Knowledge Sharing Platform, was designed. Its public release is under preparation. The data files therein automatically include links to test method descriptions and metadata. These links also persist when data is downloaded or accessed via the application programming interface described below.

The harmonized data management steps described above provide compliance with the FAIR principles [Finable, Accessible, Interoperable and Re-usable (Reiser et al. 2018)], and allows the automatic access of data at all relevant places in the EU-ToxRisk Knowledge Sharing Platform. A substantial part of this is based on the integration between BioStudies and the ToxDataExplorer, with the latter developed by Edelweiss Connect (https://www.edelweissconnect.com/blog/edelweissdata). The ToxDataExplorer interface allows users to interactively configure a uniform resource identifier for retrieving data via an application programming interface applying exactly the filtering specified by the user.

### Baseline variance of test methods

All data of the DMSO controls of the second biological replicate of each test method was analyzed. The raw values of the single technical replicates (*x*) on one plate were normalized to their average (µ) creating normalized values (*x*_norm_ = *x*/µ).

The standard deviation (SD) between the technical replicates was calculated and normalized to the average (µ) by calculating the relative standard deviation (RSD [in %] = SD *100/µ).

The resulting RSD (in percent of average) enables comparison between test methods. For the variance of test methods concerning negative control samples, three drugs were chosen (clofibrate, tolbutamide and sulfisoxazole) that have non-adverse effects in man despite prolongued exposure. Their known *C*_max_ in man is 449 µM for colchicine, 464 µM for sulfisoxazole and 196 µM for tolbutamide (Hardman JG 2001). We used here the two lowest test concentrations in each test (i.e. concentrations < 31.6 µM for clofibrate and sulfisoxazole and < 100 µM for tolbutamide). The data (normalized to the DMSO control) were collected from each partner and pooled for display.

## Results and discussion

### Assembly of a test battery

A panel of tests was selected to develop procedures of quality control, data processing and data banking within the cross-systems testing study of the (CSY) EU-ToxRisk project. Three sets of criteria were used to assemble the assays for CSY: (i) readiness level and throughput; (ii) use of cells representative of four target organs (target organ toxicity; liver, lung, brain and kidney) or for developmental and reproductive toxicity (DART). Some cells considered to lack particular organ characteristics were also included (HEK 293 and U-2 OS cells); (iii) the assays’ readouts should be a measure either of viability or of the activation of a signaling pathway related to target organ toxicity/DART.

Since one given cell type can be used for different test methods, the assays were grouped into “families” of related tests that used different exposure schemes or endpoints. For instance, test family #18 (HEK 293 cells) was used for two viability endpoints (LDH-release and resazurin reduction). In many cases, a test family allowed a viability and a functional readout, e.g. test #23 (UKN2) assessed neural crest cell viability and their migration capacity (functional; Fig. 2). A special case was the set of U-2 OS cell-based reporter assays, which allowed determination of viability and of 26 functional endpoints related to toxicity pathways (e.g. nuclear receptor activation or antagonism; Suppl. Fig. S4).

### Purpose of the testing program

A literature search for generic schemes that assembled all elements required for a cell-based ‘testing program on RDT and DART’ failed to find a comprehensive overview.

**Fig. 3:**
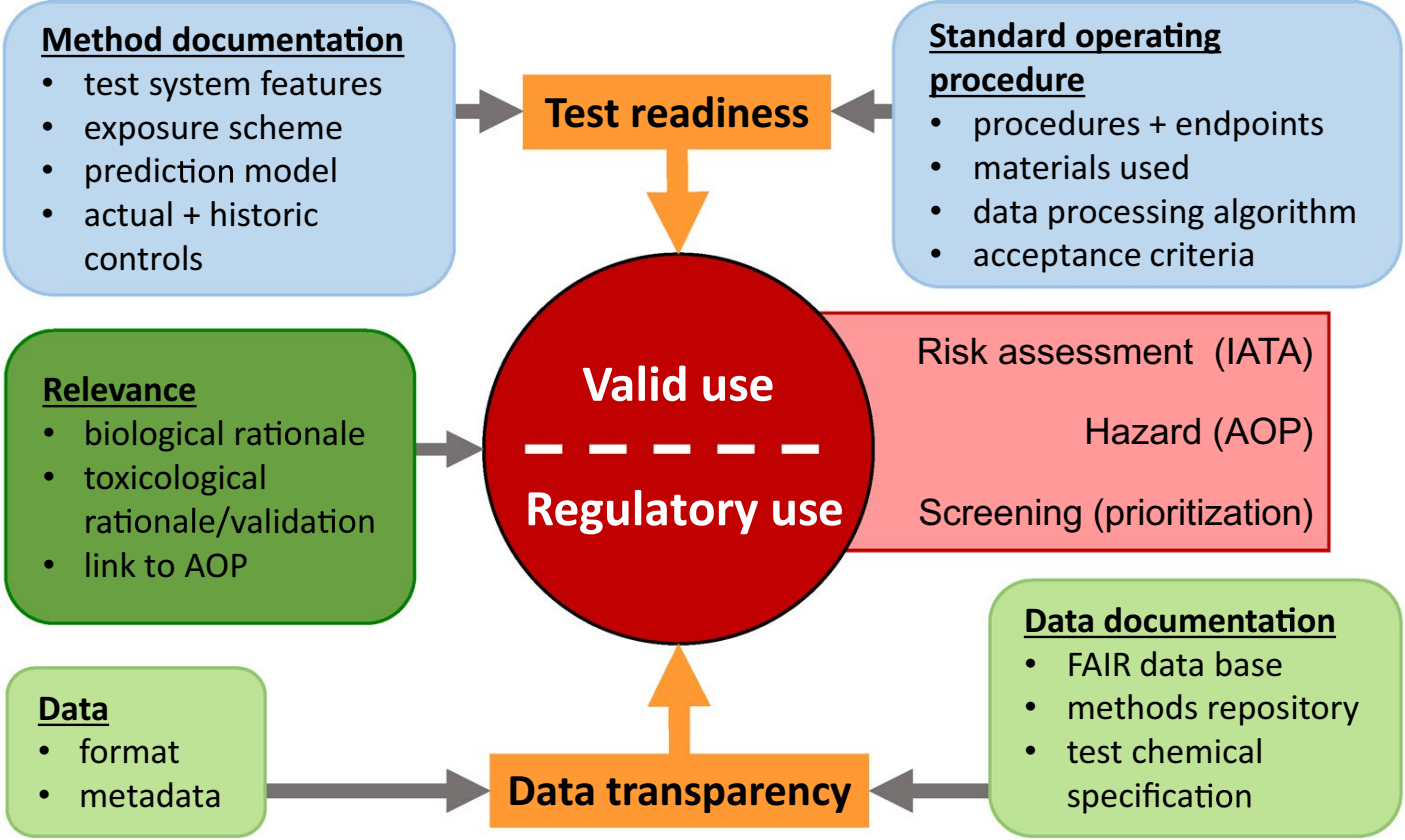
Identification of key parameters and description requirements to ensure test readiness and data transparency for regulatory use of NAM data. ‘Valid’ use, e.g. for regulatory purposes, was defined here as having a high requirement for data robustness, transparency of all procedures, and need for sufficient information on uncertainties. Three major requirements for validity were identified. First, the biological and toxicological rationale of the NAM, and the overall study objectives should be given. This may e.g. include a link to an AOP. Second, the test method applied should have been evaluated for its readiness. The latter requires complete standard operation procedures (SOPs) and a comprehensive method documentation. Third, data transparency was identified as an independent, and frequently neglected, domain to be documented. This requires the data format, and the respective metadata to be defined and documented. The data base structure needs to be designed according to findable, accessible, interoperable and re-usable criteria (FAIR), and links to the data and to the method repository need to be given. To the domain of data transparency also belongs the clear and unambiguous definition of test chemicals (e.g. SMILES and CAS numbers) including their storage, handling and toxicological background information

Therefore, we compiled the main building blocks of a comprehensive program. The core elements required were identified as (i) specification of testing purpose, (ii) description and readiness evaluation of the test methods, (iii) issues concerning the test data, and (iv) information on the toxicological and biological relevance (fit-for-purpose) of the test methods in the context of the program (Fig. 3). Moreover, we found that the selection, definition and handling of test chemicals is an essential feature.

Concerning the purpose of testing, the overarching requirement for our program was that test results were ‘valid’. We used this term to describe all situations where important human safety decisions (e.g. regulatory use) or major financial or societal questions (e.g. decisions on further development of a drug or on market introduction of a new material) depended on the data.

Examples for the broad range of applications of such ‘valid’ data include risk assessment (use of the test strategy in the context of an IATA or hazard identification (by e.g. using an adverse outcome pathway (AOP) network to guide the assembly of a test strategy). Another potential application may be the screening to prioritize problematic compounds for further testing. Depending on the exact testing purpose, details of the test strategy will need adaptation, but the main elements of the program defined here were considered broadly applicable.

The present manuscript deals with all aspects relating to the overall test program and how it was assembled. Concerning specific test results, this communication will present only a sub-set of data from one family of assays to exemplify the types of test outcomes.

### Test method documentation

Test readiness descriptions were considered here to build on two foundations: the SOP and the standardized test method description (Fig. 3). To support an exact description of the method protocol in form of a standard operation procedure (Leist and Hengstler 2018; OECD 2018a), contact was established to The European Commission’s Joint Research Center (JRC, therein EURL-ECVAM). It was agreed that SOPs would be deposited at the JRC methods’ data base DB-ALM (Roi 2006). These documents contained all commonly accepted elements of an SOP, such as detailed working procedures and descriptions of materials, instrumentation and analytical protocols.

**Fig. 4:**
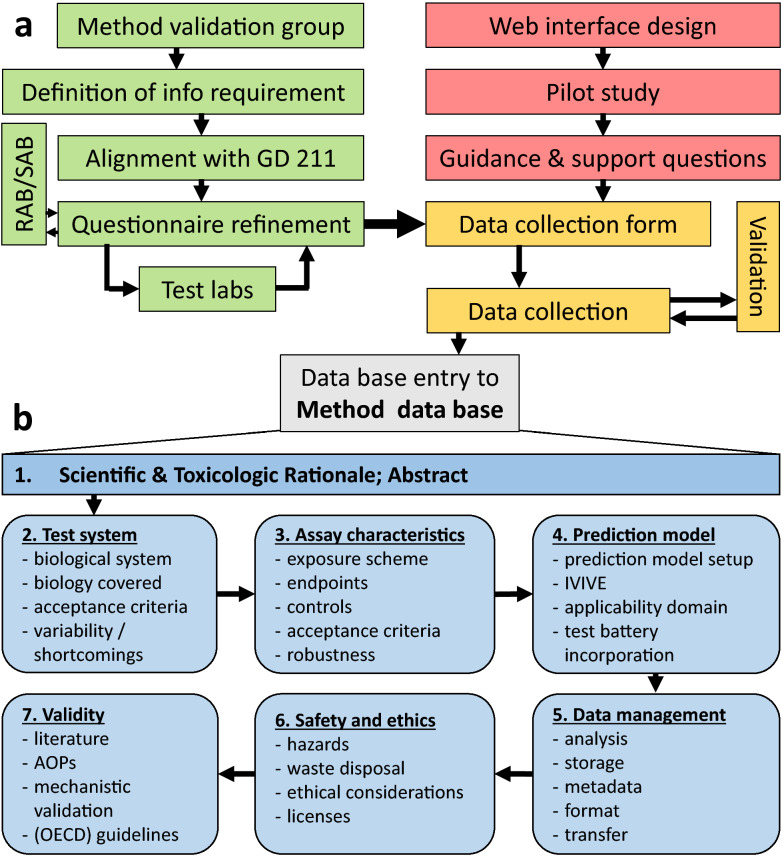
Process of establishing a method database and key information blocks documented. **a** The setup of the method database included several steps. A method validation group collected data and information that was agreed to be included in the metadata and to be documented. These were in alignment with the GD 211 of OECD to advance regulatory acceptance. The project’s regulatory and the scientific advisory board (RAB and SAB, respectively), as well as the participating test labs, contributed to refining the questionnaire for test method documentation (green). In parallel, a web interface was designed and set up to enable centralized access to the documented test methods. Within a pilot run, the upcoming issues were collected to provide guidance and support for future use (red). These two parallel approaches eventually gave rise to the data collection form. The process of data collection was constantly validated (orange). **b** An entry into the method database comprises numerous aspects of a test method. The scientific and toxicological rationale is given in the abstract. Furthermore, information about the test system, the test method/assay, its characteristics, the prediction model, data management, safety and ethics and its validity are included (color figure online)

It was considered important to complement the SOP by an overarching test method description (Krebs et al. 2019b; Leist et al. 2010, 2012a; Schmidt et al. 2017) (Fig. 3). Such a document would serve regulators to understand the method, but avoid information of limited regulatory relevance, such as pipetting steps, materials providers and instrument settings. The key elements were aligned with the OECD guidance document 211 [GD-211 (OECD 2017)] on description of non-validated test methods to be used for regulatory purposes. Multiple rounds of input came from external experts, e.g. from the project’s scientific and regulatory advisory boards, from industry stakeholders or from other, collaborating international research consortia (Fig. 4a). During pilot runs and test trials, it was found that users needed support by detailed guidance and explanations on all parts of the test methods questionnaire, and this system was again optimized with help of external experts. The final outcome was a template for the test method questionnaire (Krebs et al. 2019b), and a repository of comprehensive test method descriptions (https://eu-toxrisk.douglasconnect.com/public/) (Fig. 4b).

An SOP and a test description are not two entirely different (orthogonal) sets of information. They were produced with different users and use purposes in mind, but their contents have some overlaps. These include the definition of acceptance criteria, a comprehensive disclosure of data processing algorithms used to arrive at the assay output data (e.g. type of curve fitting, handling of outliers, etc.) and e.g. the definition of positive and negative controls. These information redundancies were welcomed, as many SOP from academically oriented labs do not follow official guidance (e.g. GIVIMP (OECD 2018a)) and may lack many of such potentially overlapping elements.

### Data handling

Data handling requirements (Fig. 3) were found to differ considerably from those of small-scale projects with mainly academic objectives. A unified format for cell-based tests was established over the course of several workshops, and all test data were deposited at European Bioinformatics Institute (EBI) in this format (https://wwwdev.ebi.ac.uk/biostudies/). The use of this professional and publicly accessible database ensured full compliance with the FAIR criteria (meaning the data are findable, accessible, interoperable and re-usable (Reiser et al. 2018).

Experience showed that some formatting demands can be so resource-requiring, that this may lead to compliance issues in a large consortium of independent partners. It is likely that a consistent deposition of data does not work if this is not supported by a suitable infrastructure and countermeasures (to meet compliance issues). Such activities include format and data base definition before project start, communication of such structures with buy-in by the users, providing interconversion scripts and easy-to-use interfaces, automated data format validation, as well as some manual curation and quality assurance efforts.

**Fig. 5:**
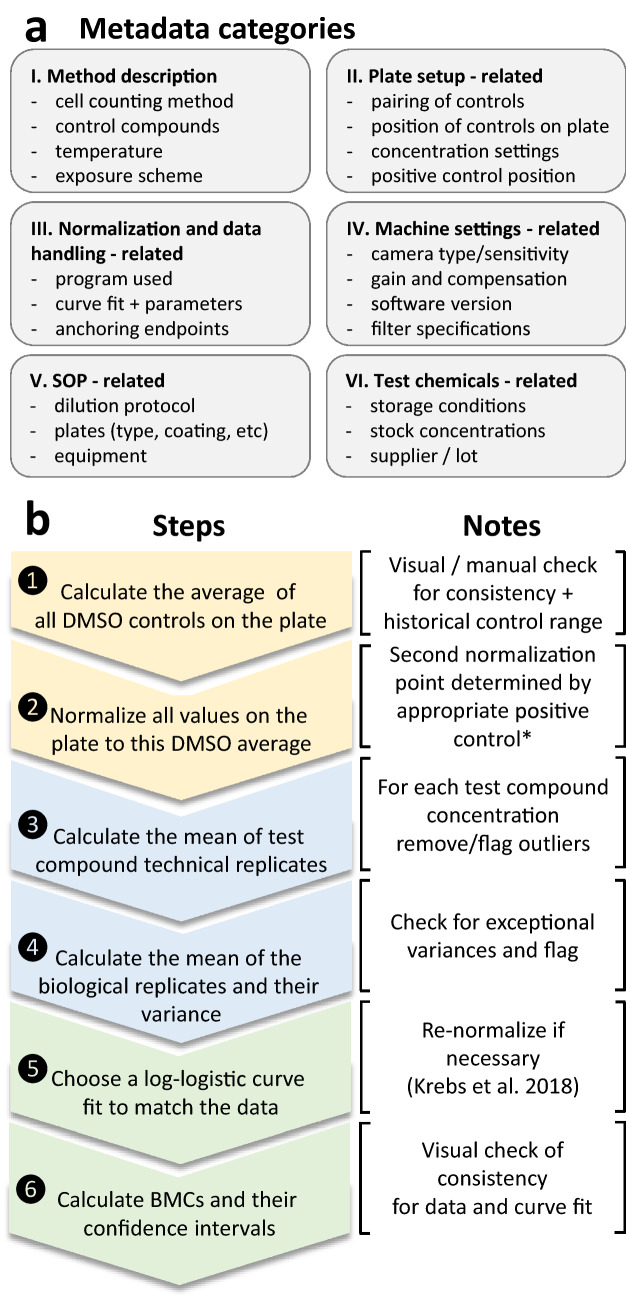
Derivation of summary data and documentation of respective metadata. **a** Overview of the types of metadata considered relevant in this study. **b** Procedure to get from raw data to summary data. *BMC* benchmark concentration

To address some of these issues, a multi-disciplinary data handling group was formed (contribution by data producers, data base specialists and data processing experts) that analyzed the projects data handling procedure and implemented problem solutions. It became clear that the academic level data handling (e.g. using Excel sheets) is error-prone. Typical problems identified are copy-paste errors, typing errors, automated format conversions by the spreadsheet program (comma recognition, interconversion of numbers to dates, …) as well as loss of information (e.g. on laboratory error flags or on identified outliers) during the handling steps. A second source of error was the association of data with their metadata (Fig. 5a). Typical examples here are (i) failures to report essential metadata (e.g. coupling of negative controls to certain data sets, positioning of samples on plates, experimental variations, links between different data sets, etc.) and (ii) copy-pasting of metadata sets without adaptation to actual experiments.

### Data processing

A further important issue of data handling was the definition of procedures to convert raw data to summary data, e.g. EC_50_ values (Fig. 5b). Here, we defined normalization procedures (Krebs et al. 2018), and agreed upon rules for curve fitting. Even with such factors being standardized, further manual (operator) input was neccessary to combine data sets (e.g. various endpoints from one given test), to update versions or to deal with problematic data sets (e.g. failure to fit curves).

The data handling experts of the project considered various strategies to ensure high-quality conversion of raw data to final summary data outputs. The highly automated and standardized approach taken e.g. by the Tox21 program/ToxCast (Richard et al. 2016; Thomas et al. 2019) was considered to rely too much on automated algorithms (vs. expert knowledge of data producers). However, it was also clear that leaving everything open to the individual data suppliers (project partners in 20 different laboratories) would cause inconsistencies. Therefore, we took a compromise approach by defining some key procedures, such as the routines for curve fitting, normalization and outlier handling (Krebs et al. 2018) and the procedures for deriving benchmark concentrations (BMCs) (Krebs et al. 2019a). The most effective quality control procedure found was to require from all data producers visual checks of graphically-represented data sets for mislabels, outliers, meaningfulness of curve-fits and consistency of summary data with the overall trend of data points (within a given data set and for different endpoints from one assay). This procedure was found to be necessary and efficient for a project producing dozens to hundreds (not thousands) of data sets. At this relatively low throughput, we considered expert knowledge to be better suited for the handling of problematic cases than fully automatic approaches.

### Fit-for-purpose test method readiness evaluation

As the EU-ToxRisk project planned for many NAM-based case studies, we explored here how the readiness of a given assay for use in one of these studies may be assessed.

A more recent perspective on validation is that the activities should focus on demonstration of a fit-for-purpose level for a given application (Bal-Price et al. 2018; Fritsche et al. 2017; Hartung et al. 2013; Judson et al. 2013; Whelan and Eskes 2016). We followed this line of reasoning and tested an evaluation scheme on four exemplary methods. Our goal was to evaluate a tool that gives a relatively quick overview of a method readiness status. A second objective was to exemplify the principle and application of readiness scoring within a running project. The selected assays differed clearly in their readiness levels.

**Fig. 6:**
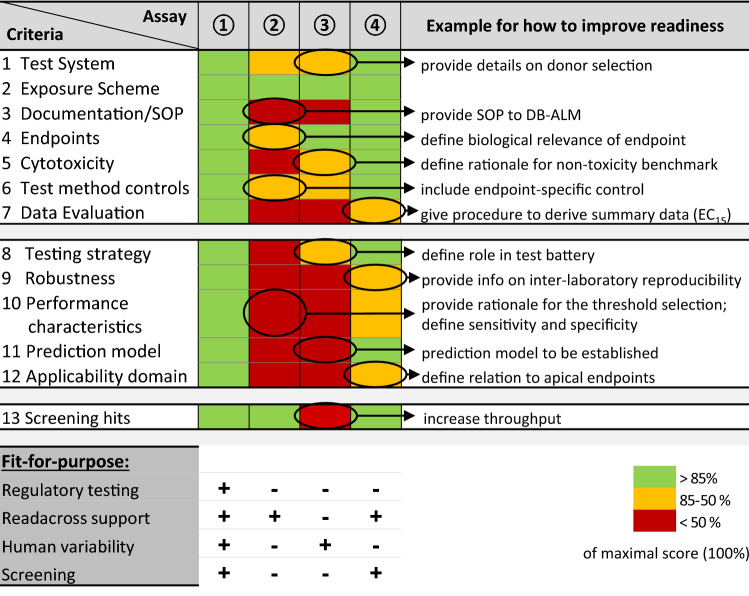
Examples for fit-for-purpose test method evaluation. Four assays of the case study were selected to exemplify the process of test readiness evaluation according to the criteria defined in a recent publication (Bal-Price et al. 2018). Thirteen different categories were scored, each of them having multiple sub-items. The summary scores of each main category were normalized to the maximum possible score. The result was indicated in green (high score), yellow, and red (low score). For instance, robustness (category 9) was high for test 1, low for tests 2 + 3 and intermediate for test 4. The first 7 categories deal usually with an earlier phase of test development (e.g. definition of the exposure scheme and endpoints), categories 8–12 require usually more extensive work (e.g. setup of a prediction model or definition of the applicability domain); the 13th category deals with special requirements arising from high-throughput screening. Several examples are given how test readiness may be improved in a given category. For instance, information on donor selection criteria may be missing for a test system based on human primary cells, or the data evaluation strategy may be incompletely described. Below the scoring table, four example applications for test methods are given, and + signs indicate whether the assay above may be suitable for this test purpose. These purely theoretical examples are meant to indicate that each test is ready for some application, but only a test with highest readiness level in all categories is useful for all different purposes. Scoring was performed by two independent experts, based on the information in the test method description. The scores were averaged, when they differed less than 20% or a third scorer was added in the few (< 10%) cases of larger discrepancies. Assay 1 was the CALUX-ER agonist assay, 2 was the RPTEC assay, 3 was the PBEC-ALI assay and 4 was UKN2. Note that the scoring was done to exemplify the procedure, not to rank assays. The scores are likely to have changed for assays, since they were scored in the year 2017 (color figure online)

Thirteen test parameters (e.g. documentation level, performance characteristics or suitability for high throughput screening), with altogether 62 sub-items (Bal-Price et al. 2018) were scored (Fig. 6).

The CALUX^®^ estrogen receptor agonist assay received top scores for all thirteen categories This outcome is in good agreement with the fact that the assay underwent full validation earlier. The UKN2/cMINC test method (neural crest cell migration assay) scored high on 9 categories and medium on the other four. The readiness level found here is consistent with the fact that the assay has been extensively used for screening e.g. for the national toxicology program of the USA (NTP) or EFSA, and several publications on test parameters are available (Nyffeler et al. 2017a, b, 2018). Although not suitable for some regulatory fields, such an assay may be used for non-regulatory decisions or screening programs.

Two other tests showed lower readiness scores, reflecting their more academic level of use. The detailed evaluation scheme used here showed that this may not be due to a lower quality of such tests, but because test documentation did not match regulatory expectations (e.g. SOP not deposited at a curated data base, or data processing not clearly indicated). Nevertheless, such tests still have a sufficient readiness levels for specific questions, such as providing mechanistic information, or giving information on human variability (using primary cells from various donors). Moreover, if their robustness is documented formally in the near future, their application in support of read-across cases can be envisaged.

For EU-ToxRisk, it is important to optimize assay readiness levels during the project, e.g. with a perspective of using the tests in a commercialization platform. This case study (CSY) has indicated a tool that can define baseline readiness levels at project start and also follow changes over the project.

In summary, we demonstrated that the “fit-for-purpose test evaluation tool” allows a differentiated (multi-parameter) overview of test readiness. It may be useful within heterogeneous research consortia, but also for communication between test providers and potential customers. Moreover, it may be considered as a tool to judge the data that are used for building AOP, as these commonly are derived from a very heterogeneous and broad panel of assays in multiple different laboratories.

### Selection and specification of compounds for cross systems testing

**Fig. 7:**
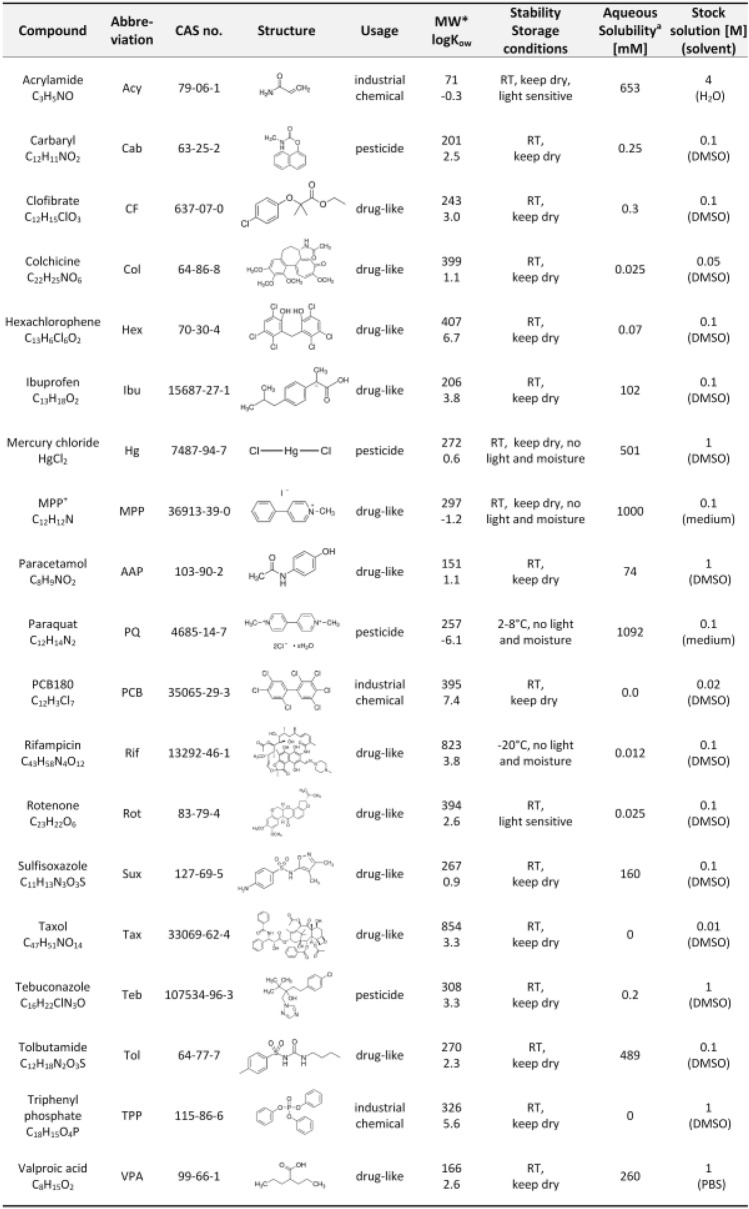
List of compounds tested in this study (CSY). Information of physiochemical properties included the molecular weight (MW, in Dalton), the lipophilicity, expressed as the logarithm of the octanol–water distribution constant (*K*_ow_), and information on preparing stock solutions. ^a^Solubility at pH 7.4. RT = room temperature. logP and aqueous solubility were derived using the Chemaxon software. Physiochemical properties derived from EPI-suite were used in calculations

A set of 19 compounds was selected to be run through all tests, so that procedures related to compound handling, and data processing could be refined. Moreover, this pilot run allowed for verification/re-adjustment of basic information on test method performances and throughput. The test panel included drugs (e.g. paracetamol, rifampicin, taxol, colchicine and valproic acid), pesticides (e.g. carbaryl, rotenone or paraquat) and other well-characterized chemicals (acrylamide, PCB180, triphenylphosphate hexachlorophene, mercury chloride, methyl-phenyl-pyridinium (MPP^+^) and tebuconazole). Four compounds with very low target organ toxicity (clofibrate, tolbutamide, ibuprofen and sulfisoxazole) were included as potential negative controls for viability assays (Fig. 7). This process led to a number of learnings that are summarized here and can be used to streamline later case studies:

(i) Compound specification and identity: common names are not sufficiently defining; at least CAS numbers should be given; ideally, an even more defining chemical descriptor (SMILES, InChI) should be considered.
(ii) Even an exact chemical identifier may not be sufficient, as the same main compound may be offered at different purities, or with certain batch variations. We opted for centrally purchasing the compounds and to distributing them to the partners from one single source.
(iii) Compound management: even with a single distributor there can be large variability for some compounds, if they are not chemically stable, if they tend to aggregate, if they are light-sensitive, etc., or if there are no clear instructions before starting a case study on how to prepare stocks, handle and store aliquots, and what specific precautions to consider when handling (e.g. diluting, sterile filtering, etc.) the chemicals. A particularly important point is information on solubility, to avoid artifacts in dilutions and testing (Fig. 7). All compound management information was included for this study in a shared document. Such a procedure is key to all collaborative studies (e.g. ring trials for validation). Experience has shown (this project included) that this issue tends to get neglected, as it is neither covered by standard test method descriptions nor by many test SOPs. Some information on this (supplier, batch, storage temperature, stock solution) are included in the EU-ToxRisk data file format. In parallel, a data-independent access of this information is advisable.
(iv) Compound classification: Several types of information are required for test compounds. First, the basic physicochemical properties (e.g. lipophilicity (logP) or volatility (Henry’s constant) represented important input for several in silico tools. For this study, the solution was to collect it in a project chemical list, deposited and updated at the EBI. A lesson from this pilot study was that it is useful to expand this list of basic features by parameters that are important for biokinetics considerations and IVIVE. These comprise protein binding and metabolic stability in hepatocyte or microsome assays. As second category of information, the toxicological characterization, is very important. We found that such datawere particularly needed for a test set of compounds to be used to characterize assay performance.

For each chemical, information should be provided for which types of toxicities (target organs) it is to be considered as a positive control or a negative control. This should be supplemented with information on which concentration is expected to result in toxicity and up to which concentration no toxicity is expected.

### Consideration of biokinetics

**Fig. 8:**
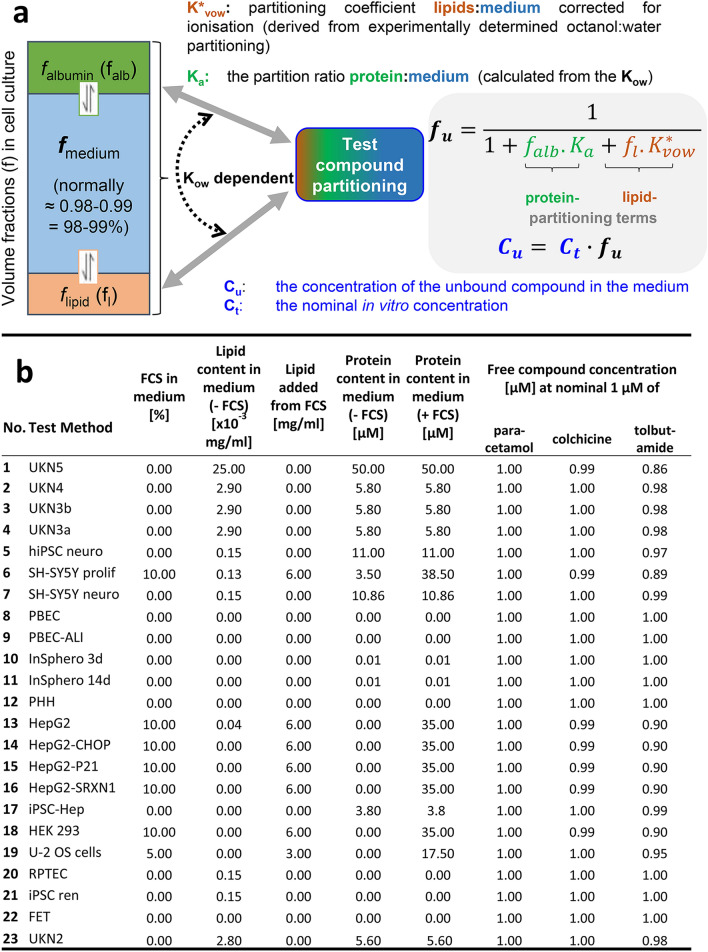
Documentation of medium compositions and estimation of free compound concentrations. **a** A model is presented that assumes that a test compound distributes to three different fractions of cell culture medium, dependent on its *K*_ow_ (octanol–water distribution coefficient). Note, that fractions are drawn here out of scale, and strictly separated. In practice, the aqueous medium comprises the largest volume fraction, and the other components (lipid and protein) are interspersed. Nevertheless, their volume can be calculated, based on their specific weight and the known amounts. This means that the volume of the protein fraction (*f*_alb_) and of the lipid fraction can be calculated, if medium composition is known (Fisher et al. 2019). With this information available, the free drug concentration can be calculated. **b** Composition of different media used for the test systems of CSY. The last three columns indicate the free compound concentrations in the different cell culture media of the test systems. Paracetamol was chosen as drug with low protein binding (15%), while colchicine (40%) and tolbutamide (95%) are known to be bound to protein to a higher percentage. For the overview table, we assumed that 100% FCS contain 346 µM albumin and ~ 6000 mg/l lipid (Lindl 2002). Free compound concentrations were calculatedas as described (Fischer et al. 2017; Fisher et al. 2019). Information on % protein binding was taken from the DrugBank data base and literature (Chappey and Scherrmann 1995; Wishart et al. 2006)

One crucial aspect of the use of NAM for hazard prediction is a conversion of in vitro points-of-departure (PoD, concentration marking the toxicity threshold) to in vivo doses in an IVIVE procedure. One fundamental input to IVIVE, but also for the comparison of test data among different test systems (some using serum, some serum-free) is the free drug concentration (not bound to protein or lipid). We adapted here an approximation formula (Fisher et al. 2019) that allows an experimenter to estimate free drug concentrations. This formula uses logK_ow_ as a predictor for lipid and protein binding, so that no further experimental data are required (Fig. 8a). All required information was compiled from the standard test chemical descriptions and the methods descriptions. The latter contains a paragraph on the lipid and protein content of the medium used. A synoptic compilation of these background data showed relatively large heterogeneity across test methods, with the amount of serum added playing the largest role (Fig. 8b). To exemplify the effect of various cell culture media, calculations were performed for three test compounds with known high, medium and low protein binding. For paracetamol (low protein binding), the free concentration was in all cases the same as the nominal test concentration. For the strong protein binding drug tolbutamide (approx. 95% protein bound in human plasma), the free concentration was 86–100% of the nominal concentration. For most media, there was < 5% difference of free and nominal concentration. This example shows that the nominal concentration is a sufficiently good concentration metric to express toxicity thresholds (PoD) for compounds in this hydrophobicity range. The situation may change when testing is performed in entirely different concentration ranges, or with the use of media with particularly high protein and lipid contents. Also, for some of the extremely hydrophobic compounds (e.g. PCB180), additional effort would be required, such as measurements of the plastic adsorption (Nyffeler et al. 2018).

### Test method baseline variation

**Fig. 9:**
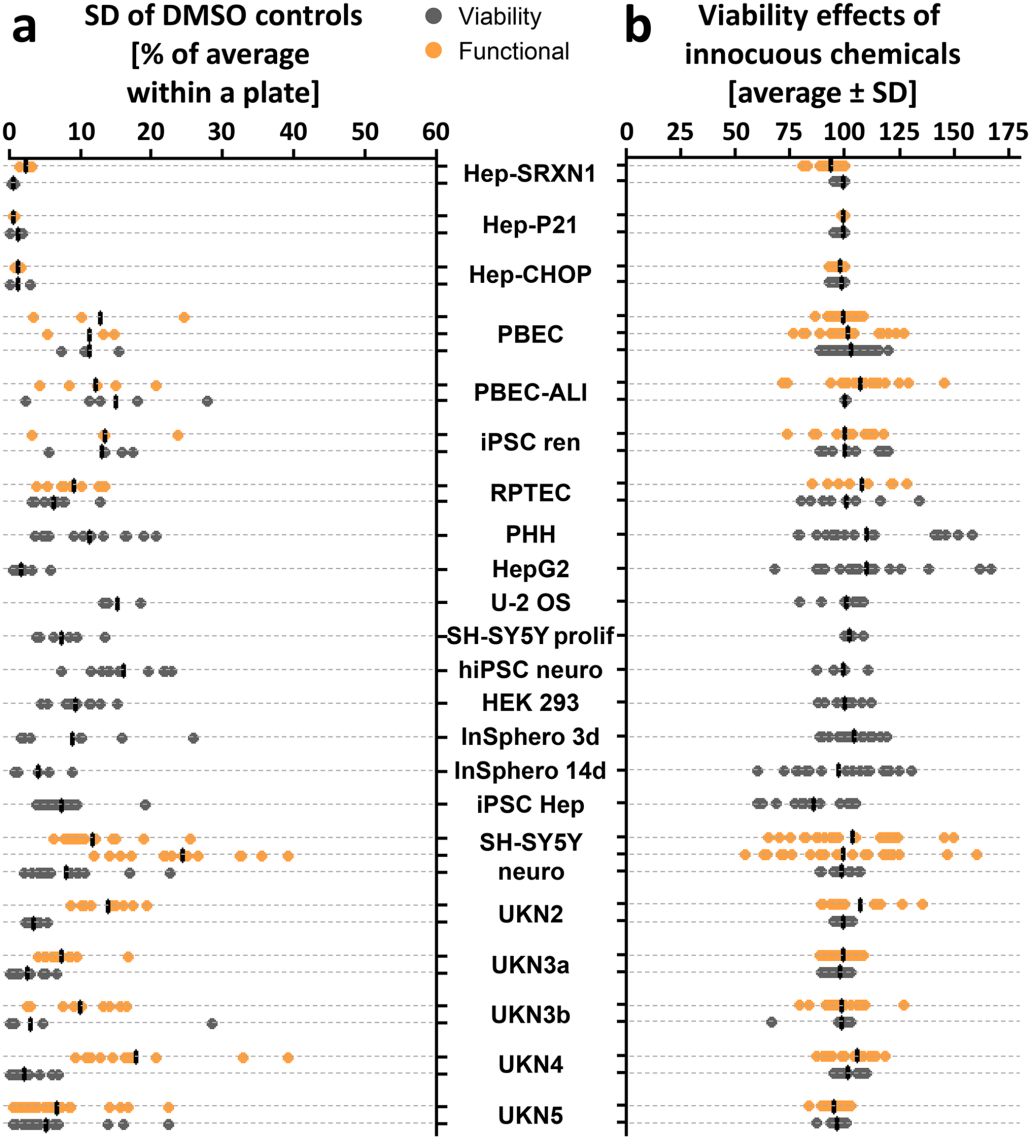
Characterization of the baseline variation (assay noise) of the NAM panel. **a** Variance of DMSO controls controls across different test methods. Each data point represents the standard deviation between technical replicates on the same plate, expressed as percent of average. The line indicates the average. **b** Variance of negative control compounds across test methods. To depict the test variance in treated samples, normalized data of the two lowest concentrations of three negative controls (clofibrate, tolbutamide and sulfisoxazole) in each test system are shown. *SD* standard deviation

With the overall testing strategy established, it also became interesting to look at the basic robustness of the 23 assays under real testing conditions. Such information can be an essential parameter for hit definition (e.g. when positive responses are defined by the noise of negative controls) (Delp et al. 2018; Dreser et al. 2019; Hsieh et al. 2019; Krug et al. 2013). We therefore determined the relative variation of solvent controls for 37 test endpoints (22 standard viability tests plus 15 functional endpoints). For all viability assays, the average variation (considering several assay plates) was < 15%, and only one out of the 37 endpoints had a coefficient of variation > 20%. For most test systems, the functional endpoint(s) showed more variation than the simple viability endpoint (Fig. 9a), but remained ≤ 20% (Suppl. Fig. 5). We also investigated the data for three non-cytotoxic negative controls (sulfisoxazole, tolbutamide, and clofibrate). The average signal from these chemicals showed 100% viability or function, and the spread was mostly between 80 and 120% of solvent control data. However, some assays showed considerable deviation (up to 50%) for some of the individual measurements (Fig. 9b).

Often, basic test parameters, such as the noise of negative controls or signal–noise ratios are determined in specific experiments dedicated to this objective. An alternative approach, chosen here, was to extract the information post-hoc from a large set of screening data. Our strategy is likely to indicate a higher variation, but it also has the advantage that such information is obtained under “real-life” test conditions and thus appears to be most relevant.

### Pathway response profiling of test chemicals in the U-2 OS reporter cell lines battery

As an example, of actual test data, we selected the CALUX^®^ assay family based on reporter constructs in U-2 OS cells. These tests altogether provide 27 endpoints. Most of them indicate agonism or antagonism of nuclear receptors (e.g. estrogen receptor, androgen receptor, thyroxid receptor, arylhydrocarbon receptor or the glucocorticoid receptor). They also cover some stress/signalling pathways (e.g. p53, Nrf-2 or AP-1). These assays were selected for several reasons: (i) the results provide additional background characterization of our test compounds by indicating AOP molecular initiating events and developmental toxicity liabilities (van der Burg et al. 2015a); (ii) the data matrix generated from these assays optimally exemplifies the problem of cytotoxicity, when functional assays are used; (iii) it also exemplifies the general data structure resulting from such a test battery with some typical problems to be dealt with: e.g. no effects until maximal test concentrations; (iv) Dealing with the whole battery (yielding several hundred endpoints for the compound set tested) will require a separate follow-up manuscript.

**Fig. 10:**
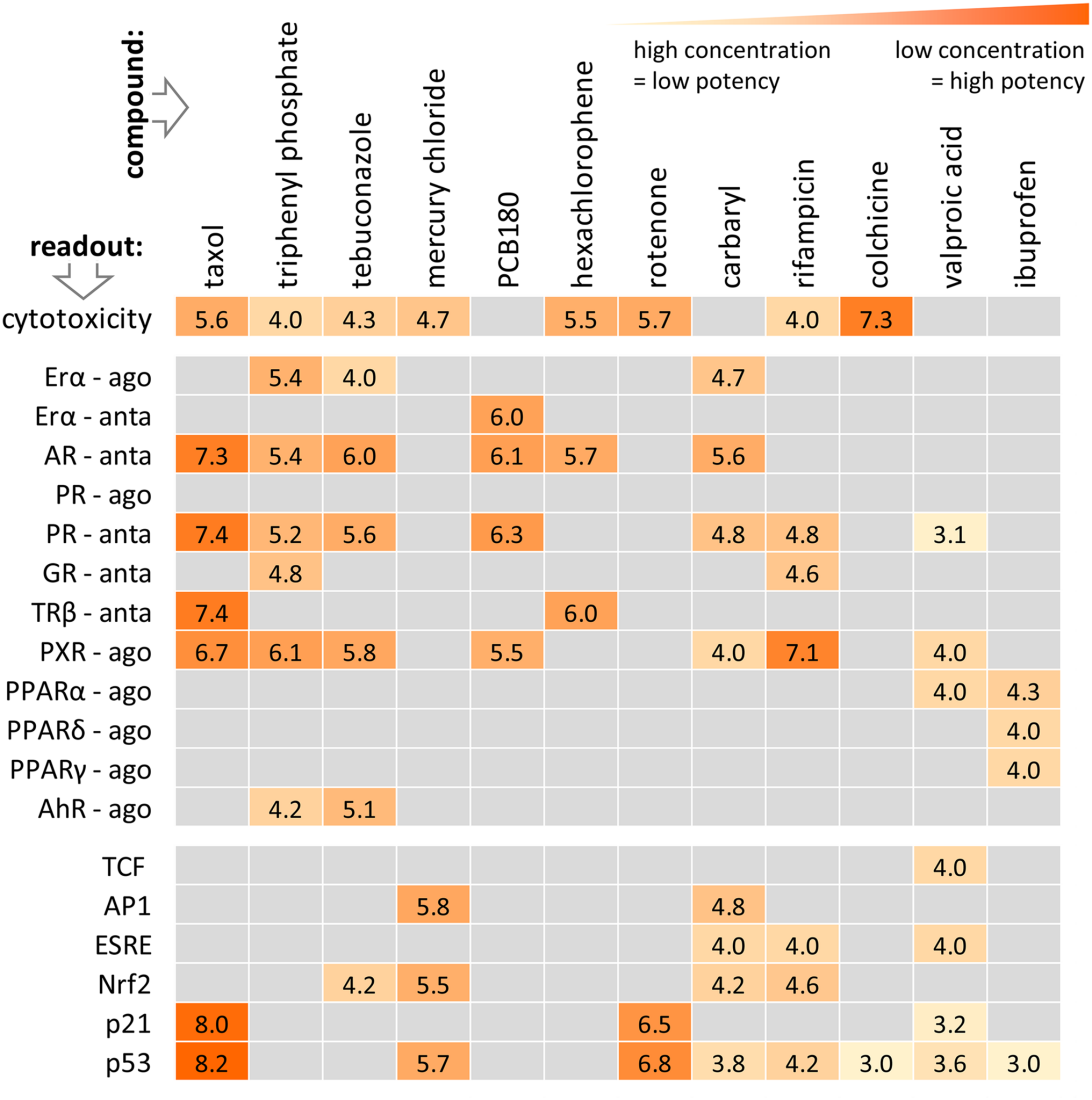
Profiling of test chemicals in the U-2 OS reporter cell lines battery. Compounds were tested at 13 concentrations (ranging from 4 to 10 [− log_10_(M)], respectively 100 µM to 0.1 nM) in the CALUX^®^ (Chemical Activated Luciferase gene eXpression) reporter gene assays of BioDetection Systems (Netherlands) in U-2 OS cells. After 24 h exposure, luciferase induction was quantified and concentration-reponse curves were modelled. The data displayed are the respective assay PoD given in − log(M). For instance, 6.0 for tebuconazole in the AR-anta assay means that its PoD was 1 µM. The exact description of the CALUX^®^ assay endpoints and the according PoDs are given in Suppl. Fig. 2. Data are means from 3 assay runs. Grey: no effect observed. Orange: concentration of PoD [–log(M)]. ago = agonist. anta = antagonist. The following assays were run, but they are not included in this display as there was no response: AR, PR, GR, RAR, LXR, Hif1α, NFκB. The following compounds had no effect, and are therefore not shown: acrylamide, MPP^+^, paracetamol, sulfisoxazole, clofibratez (color figure online)

Some exemplary compound responses in the CALUX^®^ battery were as follows. In general, activation of the receptor- or stress pathway-mediated assays was observed at concentrations ~ 10–100 × lower than the cytotoxic concentration. Taxol was the most potent compound in this study; it was active on several assays at concentrations in the lower nanomolar range, which is at least two orders of magnitude lower than most other compounds tested. It was cytotoxic in this cell system at 5.6 (note that we use a unified data format of –log(M); 5.6 corresponds to about 2.5 µM). Taxol very specifically antagonized three nuclear hormone receptors at 7.4 (below 100 nM), which suggests that this compound has endocrine activity. Additionally, taxol was found to activate expression of the p53 tumor suppressor protein at 8.2 (< 10 nM), which reflects the compound's pharmaceutical action as a microtubule stabilizer. The ability to act as antagonists on the androgen- and progesterone receptor was observed for several of the compounds, often in combination with agonistic action on the estrogen receptor (ERa-ago). Such a profile is often observed for endocrine active compounds. Triphenyl phosphate, PCB180, hexachlorophene only activated nuclear hormone receptor related assays, while for example rifampicin and carbaryl additionally activated several stress pathway related assays. HgCl_2_ and rotenone, in turn, only activated stress pathway related assays (oxidative stress, cell cycle control and DNA damage), but no nuclear receptors. Ibuprofen activated all three isoforms of the peroxisome proliferator activated receptor (PPAR), as has been described previously for several NSAIDs (Puhl et al. 2015). Colchicine was the only compound which was cytotoxic at very low concentration (50 nM), but did not significantly activate any of the assays tested (Fig. 10).

Altogether, the data showed that the test set represents a wide range of cytotoxic potencies (> 4 log steps). This knowledge is important, as single (fixed) concentration testing may not identify the toxicity of low-potency compounds such as valproic acid (VPA). Moreover, cytotoxicity anchoring informs on whether functional test hits may be caused by indirect/cytotoxic effects (Judson et al. 2016).

## Conclusion and outlook

We have used this case study to test and refine a general strategy for using a panel of assays provided by different laboratories. Several issues became only evident during this study, and several rounds of optimization were required to arrive at the final procedures disclosed here. We considered input not only from those directly concerned with experiments and data handling, but also from potential external stakeholders interested in the assays, as well as published experiences of others (Beger et al. 2019; Stephens et al. 2018; Viant et al. 2019).

One of our most important advances was the template for a comprehensive methods description, and a related database for the methods of this study (Krebs et al. 2019b), and this achievement of the CSY has been used subsequently to document methods in read-across (RAx) case studies (Escher et al. 2019). The regulators reviewing the case studies found the transparent disclosure of all methods very important, and they suggest the RAx studies to be submitted to the OECD as examples for good practice. It is planned that these case study documents will be published in 2020 (see: OECD Chemical Safety and Biosafety Progress report No. 39 Dec 2019).

We identified four important issues that require further development: (i) using readiness criteria of test methods, as a basis for fit-for-purpose evaluations; (ii) more transparency, concerning (meta)data handling and processing, (iii) better definition and documentation of the procedures for test compound management and documentation, and (iv) clear definition of study procedures objectives before initiation of the study, ideally documented in a traceable way (pre-registration as common in physics or for clinical studies).

We hope that the disclosure of this study strategy and of the problems and issues encountered during CSY will aid further progress in the field of NAM-based toxicity testing. They may be particularly useful, when tests from multiple suppliers, with different background and possibly heterogeneous readiness levels are combined to solve a toxicological question. More importantly, we are convinced that this strategy description and its further development will help to make NAM data more reliable. This would make them easier to be considered and judged by regulators, and it will thus facilitate a more wide-spread use of NAM in hazard assessment.

## Electronic supplementary material

Below is the link to the electronic supplementary material.

Supplementary file1 (PDF 591 kb)

## Abbreviations

ADME: Absorption, distribution, metabolismus and elimination
AOP: Adverse outcome pathway
AR: Androgen receptor
ATP: Adenosine triphosphate
BDS: BioDetection Systems
BIOT: BioTalentum
CALUX: Chemically activated luciferase expression
cAMP: Dibutyryl 3′,5′-cyclic adenosine monophosphate
cMINC: Circular migration of neural crest cell
CNS: Central nervous system
DART: Developmental and reproductive toxicity
DMEM: Dulbecco’s modified eagle medium
DMSO: Dimethyl sulfoxide
EC: Effective concentration
ER: Endoplasmatic reticulum
ERα: Estrogen receptor alpha
ESNATS: Embryonic Stem cell-based Novel Alternative Testing Strategies
EURL ECVAM: EU Reference Laboratory on alternatives to animal testing
FCS: Fetal calf serum
FET: Fish embryo toxicity test
FN: False negative
FP: False positive
GCCP: Good cell culture practice
GD: Guidance document
GFP: Green fluorescent protein
GIVIMP: Guidance Document on Good In Vitro Method Practices
GLP: Good laboratory practice
GR: Glucocorticoid receptor
hESC: Human embryonic stem cells
hiPSC: Human induced pluripotent stem cells
hpf: Hours post fertilization
IATA: Integrated approaches to testing and assessment
IFADO: Leibniz-Institut für Arbeitsforschung an der TU Dortmund
iPSC: Induced pluripotent stem cells
ISTNET: International STakeholder NETwork
IVIVE: In vitro to in vivo extrapolation
JRC: Joint Research Center
KUL: Katholieke Universiteit Leuven (Catholic University of Leuven)
LDH: Lactate dehydrogenase
LUMC: Leiden University Medical Center
MIE: Molecular initiating event
NAM: New approach methods
NCC: Neural crest cell
OECD: Organization for economic co-operation and development
PBEC: Primary bronchial epithelial cells
PBS: Phosphate buffered saline
PHH: Primary human hepatocytes
PNS: Peripheral nervous system
PoD: Point of departure
PPB: Plasma protein binding
PR: Progesterone receptor
PTL: Proximal tubular-like cells
QSAR: Quantitative structure–activity relationship
RAx: Readacross
Ren: Renal
RPTEC/TERT1: Renal proximal tubule epithelial cells
RSD: Relative standard deviation
RT: Room temperature
TEER: Trans-epithelial electrical resistance
TG: Test guideline
TRβ: Thyroid hormone receptor beta
UHEI: University of Heidelberg
UKN: University of Konstanz
UL: University of Leiden
VUA: Free University Amsterdam (Vrije Universiteit Amsterdam)
