# Supplementary material for: The EU-ToxRisk method documentation, data processing and chemical testing pipeline for the regulatory use of new approach methods
Source: Arch Toxicol. 2020 Jul 6;94(7):2435–61. doi: 10.1007/s00204-020-02802-6 (PMC7367925; doi:10.1007/s00204-020-02802-6)
Supplement: Supplementary file 1 — Supplementary file1 (PDF 591 kb) [file 204_2020_2802_MOESM1_ESM.pdf]

Supplementary Material for

The EU-ToxRisk Method Documentation, Data Processing and Chemical Testing Pipeline for the Regulatory Use of New Approach Methods

Alice Krebs[1,2,*], Barbara M. A. van Vugt-Lussenburg[3,*], Tanja Waldmann[1,19*], Wiebke Albrecht[4], Jan Boei[5], Maja Brajnik[6], Thomas Braunbeck[7], Tim Brecklinghaus[4], Francois Busquet[8], Andras Dinnyes[9], Joh Dokler[6], Xenia Dolde[1], Thomas Exner[6], Ciarán Fisher[10], David Fluri[11], Anna Forsby[12,20], Jan Hengstler[4], Anna-Katharina Holzer[1], Zofia Janstova[9], Paul Jennings[13], Jaffar Kisitu[1,2], Julianna Kobolak[9], Manoj Kumar[14], Alice Limonciel[13], Jessica Lundqvist[12,20], Balázs Mihalik[9], Wolfgang Moritz[11], Giorgia Pallocca[8], Andrea Paola Cediel Ulloa[12], Manuel Pastor[15], Costanza Rovida[8], Ugis Sarkans[16], Johannes P Schimming[17], Bela Z Schmidt[18], Regina Stöber[4], Tobias Strassfeld[11], Bob van de Water[17], Anja Wilmes[13], Bart van der Burg[3], Catherine Verfaille[14], Rebecca von Hellfeld[6], Harry Vrieling[5], Nanette G. Vrijenhoek[17] and Marcel Leist[1,8]

[1] In vitro Toxicology and Biomedicine, Dept inaugurated by the Doerenkamp-Zbinden foundation, University of Konstanz, 78457 Konstanz, Germany
[2] Konstanz Research School Chemical Biology, University of Konstanz, 78457 Konstanz, Germany
[3] BioDetection Systems BV, Science Park 406, 1098XH, Amsterdam, The Netherlands
[4] Leibniz-Institut für Arbeitsforschung an der TU Dortmund, Leibniz Research Centre for Working Environment and Human Factors, Ardeystraße 67, 44139 Dortmund, Germany
[5] Leiden University Medical Center, Postalzone S4-P, P.O. Box 9600, 2300 RC Leiden, Netherlands
[6] Edelweiss Connect GmbH, Technology Park Basel, Hochbergerstrasse 60C, CH-4057 Basel, Switzerland
[7] Aquatic Ecology and Toxicology Group, Centre for Organismal Studies, University of Heidelberg, Im Neuenheimer Feld 504, 69120 Heidelberg, Germany
[8] CAAT Europe, University of Konstanz, 78457 Konstanz, Germany, Steinbeis SU-1866
[9] BioTalentum Ltd., Aulich Lajos str. 26, 2100 Gödöllő, Hungary
[10] Certara UK Limited, Simcyp Division, Level 2-Acero, 1 Concourse Way, Sheffield, S1 2BJ, United Kingdom
[11] InSphero AG, Wagistrasse 27, 8952 Schlieren, Switzerland
[12] Swedish Toxicology Sciences Research Center (Swetox), Unit of Toxicology Sciences, Karolinska Institutet, Forskargatan 20, 151 36 Södertälje, Sweden
[13] Division of Molecular and Computational Toxicology, Department of Chemistry and Pharmaceutical Sciences, Vrije Universiteit Amsterdam, De Boelelaan 1108, 1081HZ Amsterdam, Netherlands
[14] Stem Cell Institute Leuven, Dept. of Development and Regeneration, KU Leuven, Stem Cell Biology and Embryology, KU Leuven, O&N IV Herestraat 49, 3000 Leuven, Belgium
[15] Research Programme on Biomedical Informatics (GRIB), Institut Hospital del Mar d'Investigacions Mèdiques (IMIM), Dept. of Experimental and Health Sciences, Universitat Pompeu Fabra, 08003 Barcelona, Spain
[16] European Molecular Biology Laboratory, European Bioinformatics Institute (EMBL-EBI), Wellcome Genome Campus, Cambridge, UK
[17] Leiden Academic Centre for Drug Research, LACDR/Toxicology, Leiden University, PO Box 9500, 2300 RA Leiden, Netherlands
[18] Switch Laboratory, VIB-KU Leuven Center for Brain & Disease Research, Department of Cellular and Molecular Medicine, KU Leuven, Herestraat 49, 3000 Leuven, Belgium
[19] trenzyme GmbH, Byk-Gulden-Str. 2 DE-78467 Konstanz, Germany
[20] Stockholm University, Department of Biochemistry and Biophysics, 10691 Stockholm, Sweden

***** These authors contributed equally

<u>Table of content</u>

		page
**SM_1:**	Table of compound solvents, supplier and catalogue no.	2
**SM_2:**	Exposure schemes of the case study test methods as part of the test method description	3 - 5
**SM_3:**	Overview of CSY test methods, including database and literature references	6 - 8
**SM_4:**	List of CALUX® assays endpoints, lowest effect levels and reference compounds	9
**SM_5:**	Variability of test endpoints (ratio of BMC and according BMCL)	10

Compound	Solvent	Supplier	Cat. no.
Paraquat	H_2O	Fluka/Sigma	36541
$HgCl_2$	DMSO	Sigma	449202
Carbaryl	DMSO	Sigma	32055
VPA	PBS	Sigma	P4543
Acrylamide	H_2O	Sigma	A3553
Colchicine	DMSO	Sigma	C9754
Rifampicin	DMSO	Sigma	R3501
Rotenone	DSMO	Sigma	R-8875
MPP^+	H_2O	Sigma	D-048
PCB180	DMSO	Fluka/Sigma	35495
Hexachlorophene	DMSO	Sigma	H-4625
Clofibrate	DMSO	Simga	C6643
Paracetamol	DMSO	Sigma	A7085
Triphenyl phosphate	DMSO	Sigma	241288
Tebuconazole	DMSO	Sigma	32013
Tolbutamide	DMSO	Sigma	T0891
Ibuprofen	DMSO	Sigma	I7905
Sulfisoxazole	DMSO	Sigma	31739
Taxol	DMSO	Tocris	1097

Supplementary Material SM_1

Krebs et al., 2020

Table of compound used in the present study, their suppliers, the solvents used, and catalogue numbers.
Cat. No = catalogue number

Test name	Test system	Exposure scheme / Endpoints	Modelled tissue / process
UKN3a	LUHMES	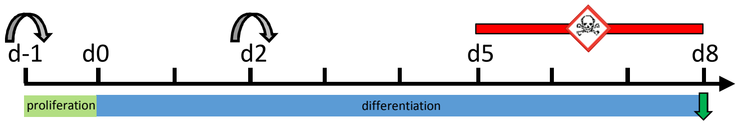	mature CNS (neurons)
Endpoint(s)		Neurite area / Viability (high content imaging)	
UKN3b	LUHMES	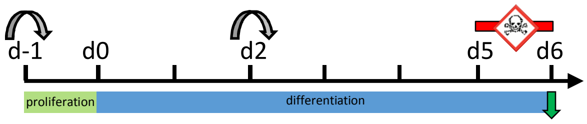	mature CNS (neurons)
Endpoint(s)		Neurite area / Viability (high content Imaging)	
UKN4 (NeuriTox)	LUHMES	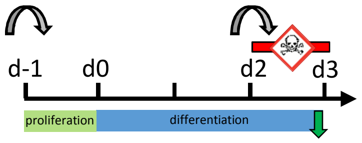	developing CNS
Endpoint(s)		Neurite outgrowth / Viability (high content imaging)	
UKN5 (PeriTox)	hiPSC	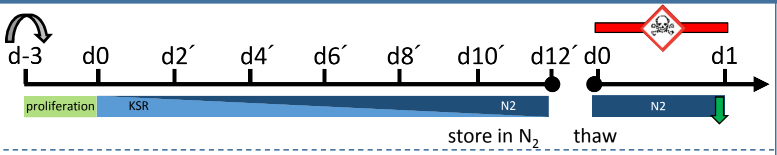	developing PNS
Endpoint(s)		Neurite outgrowth / Viability (high content imaging)	
SH-SY5Y neuro Swetox	SH-SY5Y	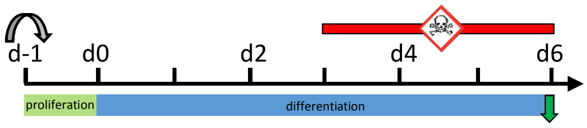	semi-mature CNS (neurons)
Endpoint(s)		Viability (ATP content) / Ca^{2+} influx	
SH-SY5Y prolif BIOT1a	SH-SY5Y	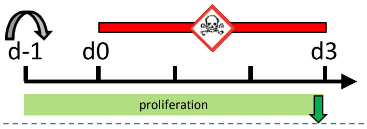	developing CNS
Endpoint(s)		Viability (ATP content)	
hiPSC neuro BIOT2a	NPC	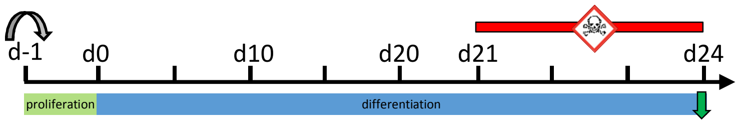	mature CNS (neurons)
Endpoint(s)		Viability (ATP content)	

	Replating
d1	Day of differentiation / day of experiment
	Compound exposure
	Day of endpoint measurement

Supplementary Material SM_2

Krebs et al., 2020

Exposure schemes of the case study test methods as part of the test method description

Test name	Test system	Exposure scheme / Endpoints	Modelled tissue / process
BIOT3a	NPC	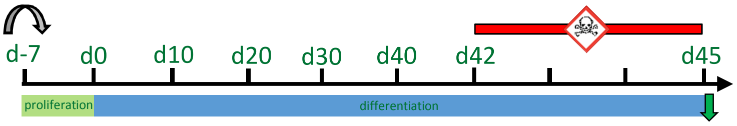	mature CNS (neurons)
**Endpoint(s)**		Viability (ATP content)	
HepG2-CHOP	HepG2 (GFP-reporter CHOP)	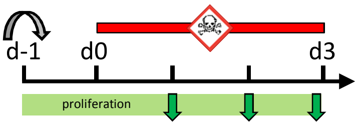	hepatocytes (stress reporter)
**Endpoint(s)**		ER stress / Viability (high content imaging)	
HepG2-P21	HepG2 (GFP-reporter P21)	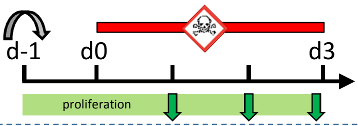	hepatocytes (stress reporter)
**Endpoint(s)**		DNA damage / Viability (high content Imaging)	
HepG2-SRXN1	HepG2 (GFP-reporter SRXN1)	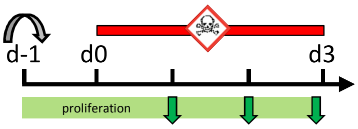	hepatocytes (stress reporter)
**Endpoint(s)**		Oxidative stress / Viability (high content imaging)	
iPSC-Hep	iPSC-derived hepatocytes	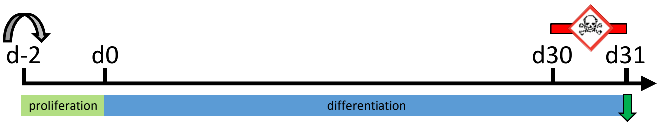	hepatocytes
**Endpoint(s)**		Neurite outgrowth / Viability (high content imaging)	
U-2 OS	U-2 OS cells	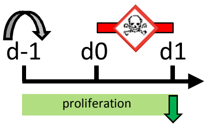	reference (pathway response)
**Endpoint(s)**		Constant luciferase expression	
RPTEC	RPTEC/ TERT1 cells	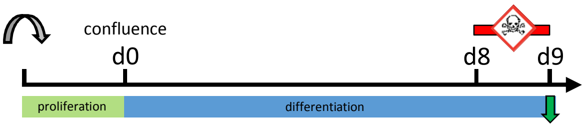	renal proximal tubule cells
**Endpoint(s)**		Viability (calcein-AM, resazurin) / cellular stress (lactate)	
iPSC ren	iPSC-derived kidney cells	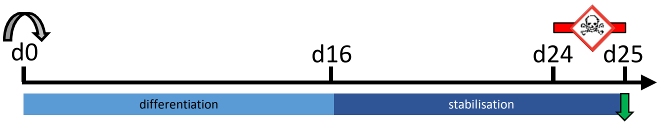	renal proximal tubule cells
**Endpoint(s)**		Viability (calcein-AM, resazurin) / cellular stress (lactate)	
FET	zebrafish embryos	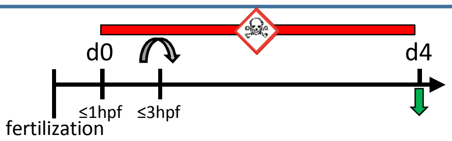	DART
**Endpoint(s)**		Viability / developmental toxicity (morphology, developmental abnormalities)	

Supplementary Material SM_2, continued

Krebs et al., 2020

Test name	Test system	Exposure scheme / Endpoints	Modelled tissue / process
UKN2	neural crest cells	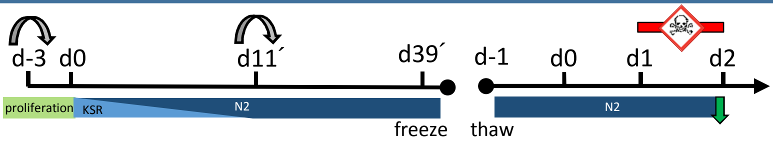	neural crest cells
**Endpoint(s)**		Migration/ Viability (high content imaging)	
HEK 293	HEK 293 cells	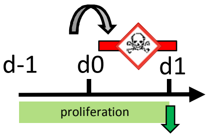	reference
**Endpoint(s)**		Viability (Resazurin assay)	
PBEC	bronchial epithelial cells	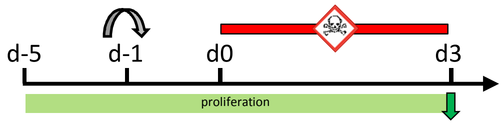	bronchial cells
**Endpoint(s)**		Proliferation/Replication (EdU staining)/ Viability (LDH assay)	
PBEC-ALI	bronchial epithelial cells	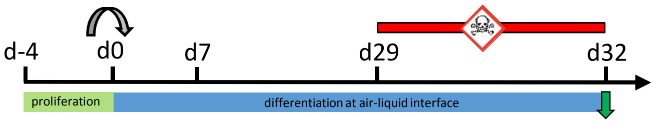	bronchial cells
**Endpoint(s)**		Proliferation/Replication (EdU staining)/ Viability (LDH assay)	
InSphero 3d	liver microtissue	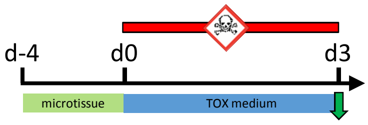	liver
**Endpoint(s)**		Viability (ATP content)	
InSphero 14d	liver microtissue	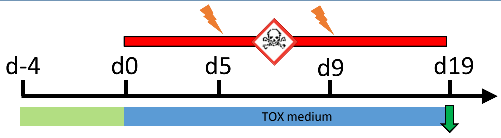	liver
**Endpoint(s)**		Viability (ATP content)	
PHH	primary human hepatocytes	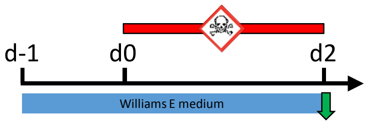	hepatocytes
**Endpoint(s)**		Morphology/ Viability (Resazurin assay)	
HepG2	HepG2 cells	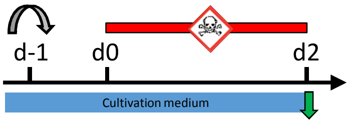	hepatocytes
**Endpoint(s)**		Morphology/ Viability (Resazurin assay)	

Supplementary Material SM_2, continued

Krebs et al., 2020

No.	Test Method abbreviation	Test System	Exposure time [h]	Readout	Database Name	Literature Reference[a]
1	UKN5	peripheral neurons	24	Cal, NA	UKN5_DART_iDRG_24h	[1]
2	UKN4	LUHMES cell line (ATCC® CRL-2927™)	24	Cal, NA	UKN4a_DART_LUH_neurite_24h	[2], [3], [4], [5]
3	UKN3b	LUHMES cell line (ATCC® CRL-2927™)	24	Cal, NA	UKN3b_NeuroTox_LUH_neurite_24h_d5<br>DB_ALM No. 196	[3], [5]
4	UKN3a	LUHMES cell line (ATCC® CRL-2927™)	72	Cal, NA	UKN3a_NeuroTox_LUH_neurite_72h_d5	[3], [5]
5	hiPSC neuro	hiPSC-derived TD21 neuronal cells	72	ATP	BIOT2a_Neuro_iPSN_Viab_72h	[32], [33], [34], [47]
6	SH SY5Y prolif	human neuroblastoma cell line (ATCC® CRL-2266™)	72	ATP	BIOT1a_Neuro_SH_Viab_72h	[28], [29], [30], [31]
7	SH SY5Y neuro	human neuroblastoma cell line, institutional subclone	72	ATP, Ca^{2+}	Swetox1a_Neuro_SH-SY5Y_Diff_3D_ATP_Exp72h<br>Swetox2_Neuro_SH-SY5Y_Diff_3D_calcium_Exp72h	[6], [7], [28], [36], [37], [50]
8	PBEC	primary bronchial epithelial cells	72	LDH, EdU	LUMC1_Lung_PBEC_Sub_72h	[8]
9	PBEC-ALI	primary bronchial epithelial cells air-exposed culture conditions	72	LDH, TEER	LUMC2_Lung_PBEC_ALI_72h	[9], [10], [11]
10	InSphero 3d	human liver microtissues composed of multi-donor PHH and NPCs (non-parenchymal cells)	72	ATP	IS1_Liver_LiMT_Tox_3D	[12], [13]
11	InSphero 14d	human liver microtissues composed of multi-donor PHH and NPCs (non-parenchymal cells)	336	ATP	IS1_Liver_LiMT_Tox_14D	[12], [13]
12	PHH	primary human hepatocytes isolated from three different donors	48	Res, GE, Morph	IFADO2a_Liver_PHH_Cytotox_CTB_48h	[14]
13	HepG2	HepG2 hepatocellular carcinoma cell line (ATCC® HB-8065™)	48	Res, GE, Morph	IFADO1a_Liver_HepG2_Cytotox_CTB_48h	[38], [39]
14	HepG2-CHOP	HepG2 BAC-GFP stress reporter cell line	24 , 48 and 72	Cell count, PI, GFP	UL2_ERstress_HepG2_BAC-GFP_CHOP_24hTP<br>UL3_ERstress_HepG2_BAC-GFP_CHOP_48hTP<br>UL4_ERstress_HepG2_BAC-GFP_CHOP_72hTP	[15], [16], [48], [49]
15	HepG2-P21	HepG2 BAC-GFP stress reporter cell line	24, 48 and 72	Cell count, PI, GFP	UL6_DNAdamage_HepG2_BAC-GFP_P21_24TP<br>UL7_DNAdamage_HepG2_BAC-GFP_P21_48hTP<br>UL8_DNAdamage_HepG2_BAC-GFP_P21_72hTP	[15], [16], [48], [49]
16	HepG2 SRXN1	HepG2 BAC-GFP stress reporter cell line	24, 48 and 72	Cell count, PI, GFP	UL10_OXstress_HepG2_BAC-GFP_SRXN1_24hTP<br>UL11_OXstress_HepG2_BAC-GFP_SRXN1_48hTP<br>UL12_OXstress_HepG2_BAC-GFP_SRXN1_72hTP	[15], [16], [48], [49]
17	iPSC-Hep	pluripotent stem cell-derived 6x hepatocytes	24	Res, LDH		[17], [18]
18	HEK 293	human embryonic kidney cell line (ATCC® CRL-1573™)	24	Res, LDH	UKN6a_control_HEK_viability_24h	[40]
19	U-2 OS	human bone osteosarcoma cell line (ATCC® HTB-96™)	24	LUX	BDS22a_Tox_RGA_cytotox_act_24h	[19], [20], [23]
20	RPTEC	RPTEC/TERT1 cell line (ATCC® CRL-4031™)	24	Cal, Res, Lac	MUI1_Renal_RT_96w_RCL_24hb<br>VU1a_MitoTox_RT_lactate_24h<br>VU1_MitoTox_RT_resazurin_24h	[21], [22], [35]
21	iPSC ren	iPSC-derived proximal tubule-like cells	24	Cal, Res, Lac		[43], [46]
22	FET	zebrafish embryos	96	Morph, Dev	UHEI1_DART_FET_120h	[41], [42], [44], [45]
23	UKN2	neural crest cells (NCC) derived from iPSC	24	Cal, Migr	UKN2a_DART_NC_cMIGR_24h	[24], [25], [26], [27]
24	hiPSC neuro	hiPSC-derived TD42 neuronal cells	72	ATP	BIOT3a_Neuro_iPSN_Viab_72h	[32], [33], [34], [47]

Supplementary Material SM_3

Krebs et al., 2020

Supplementary Material SM_3, continued

Readout abbreviations:

ATP = intracellular ATP levels; $Ca2^{+}$ = calcium signaling; Cal = Calcein staining of living cells; Dev = developmental abnormalities; EdU = 5-ethynyl-2´-deoxyuridine staining of proliferating cells; GE = gene expression; GFP = GFP expression; Lac = lactate levels; LDH = extracellular LDH levels; LUX = luciferase expression; Migr = cell migration; Morph = (cell) morphology; NA = neurite area; PI = propidium iodide staining of dead cells; Res = Resazurin metabolisation; TEER = transepithelial electrical resistance.

Literature references:

1 Hoelting, L., et al., *Stem Cell-Derived Immature Human Dorsal Root Ganglia Neurons to Identify Peripheral Neurotoxicants.* Stem Cells Transl Med, 2016. 5(4): p. 476-87.

2 Krug, A.K., et al., *Evaluation of a human neurite growth assay as specific screen for developmental neurotoxicants.* Arch Toxicol, 2013. 87(12): p. 2215-31.

3 Stiegler, N.V., et al., *Assessment of chemical-induced impairment of human neurite outgrowth by multiparametric live cell imaging in high-density cultures.* Toxicol Sci, 2011. 121(1): p. 73-87.

4 Scholz, D., et al., *Rapid, complete and large-scale generation of post-mitotic neurons from the human LUHMES cell line.* J Neurochem, 2011. 119(5): p. 957-71.

5 Lotharius, J., et al., *Progressive degeneration of human mesencephalic neuron-derived cells triggered by dopamine-dependent oxidative stress is dependent on the mixed-lineage kinase pathway.* J Neurosci, 2005. 25(27): p. 6329-42.

6 Attoff, K., et al., *Acrylamide affects proliferation and differentiation of the neural progenitor cell line C17.2 and the neuroblastoma cell line SH-SY5Y.* Toxicol In Vitro, 2016. 35: p. 100-11.

7 Gustafsson, H., et al., *Neurofunctional endpoints assessed in human neuroblastoma SH-SY5Y cells for estimation of acute systemic toxicity.* Toxicol Appl Pharmacol, 2010. 245(2): p. 191-202.

8 van Wetering, S., et al., *Regulation of secretory leukocyte proteinase inhibitor (SLPI) production by human bronchial epithelial cells: increase of cell-associated SLPI by neutrophil elastase.* J Investig Med, 2000. 48(5): p. 359-66.

9 Boei, J., et al., *Xenobiotic metabolism in differentiated human bronchial epithelial cells.* Arch Toxicol, 2017. 91(5): p. 2093-2105.

10 Amatngalim, G.D., et al., *Basal cells contribute to innate immunity of the airway epithelium through production of the antimicrobial protein RNase 7.* J Immunol, 2015. 194(7): p. 3340-50.

11 van Wetering, S., et al., *Epithelial differentiation is a determinant in the production of eotaxin-2 and -3 by bronchial epithelial cells in response to IL-4 and IL-13.* Mol Immunol, 2007. 44(5): p. 803-11.

12 Messner, S., et al., *Multi-cell type human liver microtissues for hepatotoxicity testing.* Arch Toxicol, 2013. 87(1): p. 209-13.

13 Kijanska, M. and J. Kelm, *In vitro 3D Spheroids and Microtissues: ATP-based Cell Viability and Toxicity Assays*, in *Assay Guidance Manual*, G.S. Sittampalam, et al., Editors. 2004: Bethesda (MD).

14 Godoy, P., et al., *Recent advances in 2D and 3D in vitro systems using primary hepatocytes, alternative hepatocyte sources and non-parenchymal liver cells and their use in investigating mechanisms of hepatotoxicity, cell signaling and ADME.* Arch Toxicol, 2013. 87(8): p. 1315-530.

15 Wink, S., et al., *Quantitative high content imaging of cellular adaptive stress response pathways in toxicity for chemical safety assessment.* Chem Res Toxicol, 2014. 27(3): p. 338-55.

16 Wink, S., et al., *High-content imaging-based BAC-GFP toxicity pathway reporters to assess chemical adversity liabilities.* Arch Toxicol, 2017. 91(3): p. 1367-1383.

17 Roelandt, P., et al., *Human pluripotent stem cell-derived hepatocytes support complete replication of hepatitis C virus.* J Hepatol, 2012. 57(2): p. 246-51.

18 Roelandt, P., J. Vanhove, and C. Verfaillie, *Directed differentiation of pluripotent stem cells to functional hepatocytes.* Methods Mol Biol, 2013. 997: p. 141-7.

19 van der Linden, S.C., et al., *Development of a panel of high-throughput reporter-gene assays to detect genotoxicity and oxidative stress.* Mutat Res Genet Toxicol Environ Mutagen, 2014. 760: p. 23-32.

20 van der Burg, B., et al., *A high throughput screening system for predicting chemically-induced reproductive organ deformities.* Reprod Toxicol, 2015. 55: p. 95-103.

21 Limonciel, A., et al., *Lactate is an ideal non-invasive marker for evaluating temporal alterations in cell stress and toxicity in repeat dose testing regimes.* Toxicol In Vitro, 2011. 25(8): p. 1855-62.
22 Aschauer, L., et al., *Expression of xenobiotic transporters in the human renal proximal tubule cell line RPTEC/TERT1.* Toxicol In Vitro, 2015. 30(1 Pt A): p. 95-105.
23 van Vugt-Lussenburg, B.M.A., et al., *Incorporation of metabolic enzymes to improve predictivity of reporter gene assay results for estrogenic and anti-androgenic activity.* Reprod Toxicol, 2018. 75: p. 40-48.
24 Nyffeler, J., et al., *Design of a high-throughput human neural crest cell migration assay to indicate potential developmental toxicants.* ALTEX, 2017. 34(1): p. 75-94.
25 Zimmer, B., et al., *Profiling of drugs and environmental chemicals for functional impairment of neural crest migration in a novel stem cell-based test battery.* Arch Toxicol, 2014. 88(5): p. 1109-26.
26 Zimmer, B., et al., *Evaluation of developmental toxicants and signaling pathways in a functional test based on the migration of human neural crest cells.* Environ Health Perspect, 2012. 120(8): p. 1116-22.
27 Thomson, J.A., et al., *Embryonic stem cell lines derived from human blastocysts.* Science, 1998. 282(5391): p. 1145-7.
28 Biedler, J.L., et al., *Multiple neurotransmitter synthesis by human neuroblastoma cell lines and clones.* Cancer Res, 1978. 38(11 Pt 1): p. 3751-7.
29 Pahlman, S., et al., *Retinoic acid-induced differentiation of cultured human neuroblastoma cells: a comparison with phorbolester-induced differentiation.* Cell Differ, 1984. 14(2): p. 135-44.
30 Lopes, F.M., et al., *Comparison between proliferative and neuron-like SH-SY5Y cells as an in vitro model for Parkinson disease studies.* Brain Res, 2010. 1337: p. 85-94.
31 Xie, H.R., L.S. Hu, and G.Y. Li, *SH-SY5Y human neuroblastoma cell line: in vitro cell model of dopaminergic neurons in Parkinson's disease.* Chin Med J (Engl), 2010. 123(8): p. 1086-92.
32 Chambers, S.M., et al., *Highly efficient neural conversion of human ES and iPS cells by dual inhibition of SMAD signaling.* Nat Biotechnol, 2009. 27(3): p. 275-80.
33 Shi, Y., P. Kirwan, and F.J. Livesey, *Directed differentiation of human pluripotent stem cells to cerebral cortex neurons and neural networks.* Nat Protoc, 2012. 7(10): p. 1836-46.
34 Zhou, S., et al., *The positional identity of iPSC-derived neural progenitor cells along the anterior-posterior axis is controlled in a dosage-dependent manner by bFGF and EGF.* Differentiation, 2016. 92(4): p. 183-194.
35 Wieser, M., et al., *hTERT alone immortalizes epithelial cells of renal proximal tubules without changing their functional characteristics.* Am J Physiol Renal Physiol, 2008. 295(5): p. F1365-75.
36 Pahlman, S., et al., *Human neuroblastoma cells in culture: a model for neuronal cell differentiation and function.* Acta Physiol Scand Suppl, 1990. 592: p. 25-37.
37 Agholme, L., et al., *An in vitro model for neuroscience: differentiation of SH-SY5Y cells into cells with morphological and biochemical characteristics of mature neurons.* J Alzheimers Dis, 2010. 20(4): p. 1069-82.
38 Darlington, G.J., J.H. Kelly, and G.J. Buffone, *Growth and hepatospecific gene expression of human hepatoma cells in a defined medium.* In Vitro Cell Dev Biol, 1987. 23(5): p. 349-54.
39 Aden, D.P., et al., *Controlled synthesis of HBsAg in a differentiated human liver carcinoma-derived cell line.* Nature, 1979. 282(5739): p. 615-6.
40 Shaw, G., et al., *Preferential transformation of human neuronal cells by human adenoviruses and the origin of HEK 293 cells.* FASEB J, 2002. 16(8): p. 869-71.
41 Nagel, R., *DarT: The embryo test with the Zebrafish Danio rerio--a general model in ecotoxicology and toxicology.* ALTEX, 2002. 19 Suppl 1: p. 38-48.
42 OECD, *Test No. 236: Fish Embryo Acute Toxicity (FET) Test*. 2013.
43 Rauch, C., et al., *Differentiation of human iPSCs into functional podocytes.* PLoS One, 2018. 13(9): p. e0203869.
44 Braunbeck, T., et al., *The fish embryo test (FET): origin, applications, and future.* Environ Sci Pollut Res Int, 2015. 22(21): p. 16247-61.
45 Braunbeck, T., et al., *Towards an alternative for the acute fish LC(50) test in chemical assessment: the fish embryo toxicity test goes multi-species -- an update.* ALTEX, 2005. 22(2): p. 87-102.
46 Jennings, P., et al., *Cyclosporine A induces senescence in renal tubular epithelial cells.* Am J Physiol Renal Physiol, 2007. 293(3): p. F831-8.
47 Morrison, M., et al., *StemBANCC: Governing Access to Material and Data in a Large Stem Cell Research Consortium.* Stem Cell Rev, 2015. 11(5): p. 681-7.
48 Schimming, J.P., et al., *System Microscopy of Stress Response Pathways in Cholestasis Research.* Methods Mol Biol, 2019. 1981: p. 187-202.
49 Wink, S., et al., *Dynamic imaging of adaptive stress response pathway activation for prediction of drug induced liver injury.* Arch Toxicol, 2018. 92(5): p. 1797-1814.
50 Nordin-Andersson, M., et al., Acrylamide-induced effects on general and neurospecific cellular functions during exposure and recovery. Cell BiolToxicol, 2013. 19(1): p.43-51.

Cell line	Endpoint	Lowest effect levels	Reference compound
ERa CALUX	Estrogen receptor agonists	PC10	estradiol
ERa-anti CALUX	Estrogen receptor antagonists	PC20	tamoxifen
AR CALUX	Androgen receptor agonists	PC10	dihydrotestosterone (DHT)
AR-anti CALUX	Androgen receptor antagonists	PC20	flutamide
PR CALUX	Progesterone receptor agonists	PC10	Org2058
PR-anti CALUX	Progesterone receptor antagonists	PC20	Ru486
GR CALUX	Glucocorticoid receptor agonists	PC10	dexamethasone (DEX)
GR-anti CALUX	Glucocorticoid receptor antagonists	PC20	Ru486
TRb CALUX	Thyroid receptor agonists	PC10	3,3‘5-trioiodo-L-thyronine (T3)
TRb-anti CALUX	Thyroid receptor antagonists	PC20	deoxynivalenol
RAR CALUX	Retinoic acid receptor agonists	PC10	retinoic acid
LXR CALUX	Liver X receptor agonists	PC10	GW3965 hydrochloride
PXR CALUX	Pregnane X receptor agonists	PC10	nicardipine
PPARa CALUX	Peroxisome proliferator activated receptor agonists	PC10	GW7647
PPARg2 CALUX	Peroxisome proliferator activated receptor agonists	PC10	rosiglitazone
PPARd CALUX	Peroxisome proliferator activated receptor agonists	PC10	L-165,041
DR CALUX / AhR	Aryl Hydrocarbon receptor agonists	PC10	2,3,7,8-TCDD
Hif1a CALUX	Chemical hypoxia response	PC10	cobaltous(II)chloride
TCF CALUX	wnt/TCF pathway activation	FI=1.5	lithium chloride
AP-1 CALUX	AP1 pathway activation / cell cycle control	PC10	TPA (PMA)
ESRE CALUX	Endoplasmic reticulum stress	PC10	tunicamycin
NFkB CALUX	Activation of NF-kB pathway (immune response)	PC10	TPA (PMA)
Nrf2 CALUX	Oxidative stress	FI=1.5	curcumine
p21 CALUX	Transcription of p21 inhibitor of cell cycle progression	PC10	actinomycin D
p53 CALUX	p53-dependent pathway activation / genotoxicity	FI=1.5	actinomycin D
Cytotox CALUX	Cytoxicity	PC20	tributyltinacetate

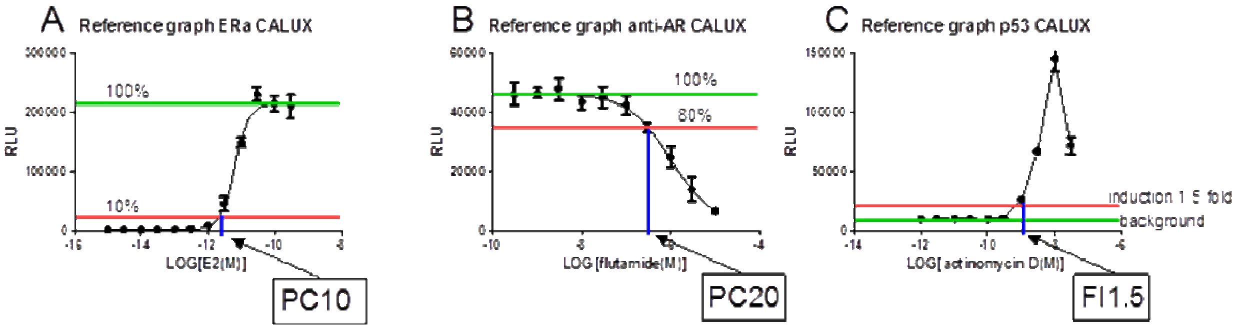

Supplementary Material SM_4

Krebs et al., 2020

Overview of CALUX assays. The synoptic table indicates what endpoint is assessed in each of the listed assays. More importantly, key information is given on how the assay endpoint is determined from the individual data points. The point od departure (PoD) if all these assays is defined by a “lowest effect level”. A typical endpoint for an agonist assay is a PC10. This is defined as the concentration of test compound giving a signal level that corresponds to 10% of the difference of the baseline (no agonist) and the maximal signal obtained with a reference agonist (as indicated in the right column of the table). a typical endpoint for an antagonist assay is a PC20. This corresponds to the concentration of a test compound that reduces the signal of the reference agonist by 20%. the right column gives examples for positive controls (=reference antagonists) used to define acceptance criteria for the assays.

PC = positive control. FI = fold induction. **PC10:** concentration where test compound causes an activation equal to 10% of the maximum effect (positive control). **PC20:** concentration where test compound causes an activation equal to 20% of the maximum effect (positive control). **FI=1.5:** concentration where test compound elicits a response 1.5-fold above background (DMSO). **(A)** exemplary graph of concentration-response-curve of receptor activation assay with PC10 as lowest effect level. **(B)** exemplary graph of receptor antagonism assay with PC20 as lowest-effect-level. (C) exemplary graph for assay with 1.5 fold induction as lowest effect level.

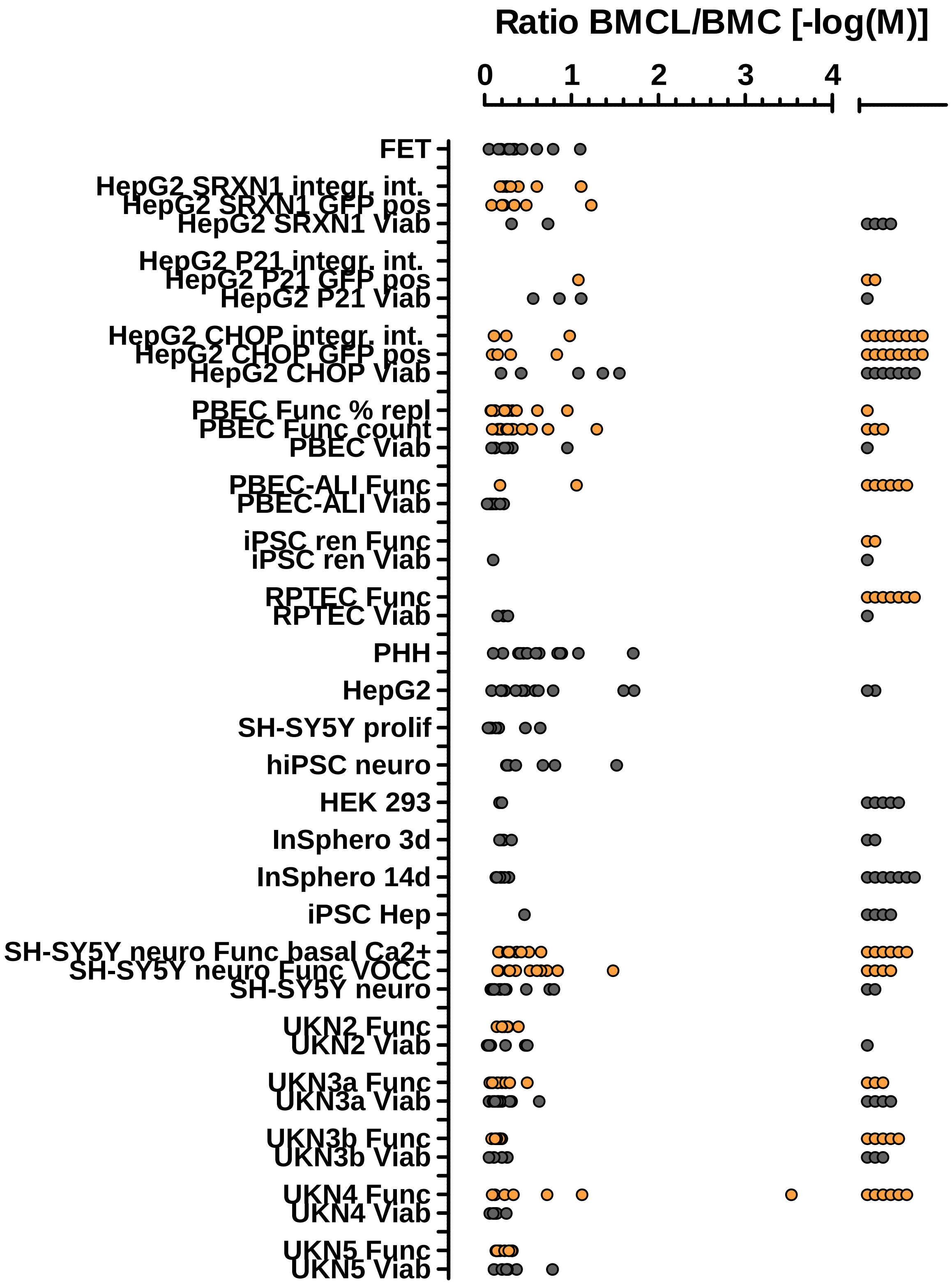

Supplementary Material SM_5

Krebs et al., 2020

Variability of tests. The BMCL as measure of test variability has been set to relation of the according BMC. The concentrations are given in –log(M) (the calculation of the ratio is hence BMCL-BMC). A ratio of 0 in logarithmic scale equals 1, a ratio of 1 in logarithmic scale equals 10. The smaller the ration, the smaller the variability of the test endpoint. 19 compounds have been tested in each test endpoint, each dot represents data from one compound. Where the BMCL could not be determined, the ratio is depicted on the right side of the axis break. Func = functional endpoint. Viab = viability
